# Investigation of Phosphonic Acids Based on Raman and Surface-Enhanced Raman Spectroscopy

**DOI:** 10.1021/acsomega.5c04862

**Published:** 2025-08-27

**Authors:** Linus Pauling F. Peixoto, Bismark N. da Silva, Regina D. E. Carvalho, Rosane A. Fontes, Luiz A. Sacorague, Tiago C. Freitas, Jussara M. Silva, Giselle M. L. L. da Silva, Monica T. da Silva, Cristano Fantini, Mariana B. Barbosa, Isabela M. F. Lopes

**Affiliations:** † Instituto SENAI de Inovação em Engenharia de Superfícies − Centro de inovação e Tecnologia CIT SENAI, Horto, Belo Horizonte, MG 31035-536, Brazil; ‡ 125096 Centro de Pesquisas, Desenvolvimento e Inovação Leopoldo Américo Miguez de Mello − Cenpes/Petrobras, Ilha do Fundão, Rio de Janeiro, RJ 21941-915, Brazil; § Departamento de Física, 28114 Universidade Federal de Minas Gerais, Belo Horizonte, MG 31270-901, Brazil

## Abstract

Phosphonic acids, such as amino tris­(methylenephosphonic acid) (ATMP) and diethylenetriamine penta­(methylenephosphonic acid) (DTPMP), are used in various applications, including scale control, water treatment, and corrosion protection. The increasing use of these compounds has raised environmental concerns due to their slow degradation, which can lead to eutrophication and the release of toxic byproducts. The detection of these compounds using surface-enhanced Raman spectroscopy (SERS) can be an interesting tool for monitoring their presence in aquatic environments. However, the vibrational characterization of these compounds has not yet been fully described in the literature. In this study, phosphonic acids ATMP and DTPMP were analyzed using density functional theory (DFT) and Raman/SERS spectroscopy. The theoretical spectra obtained were consistent with the experimental spectra, and the vibrational assignments aligned with those of organic compounds with similar structures reported in the literature. Furthermore, SERS analysis revealed bands for both compounds at concentrations up to 50 ppm (1.67 × 10^–4^ mol L^–1^ for ATMP and 8.72 × 10^–5^ mol L^–1^ for DTPMP).

## Graphical abstract

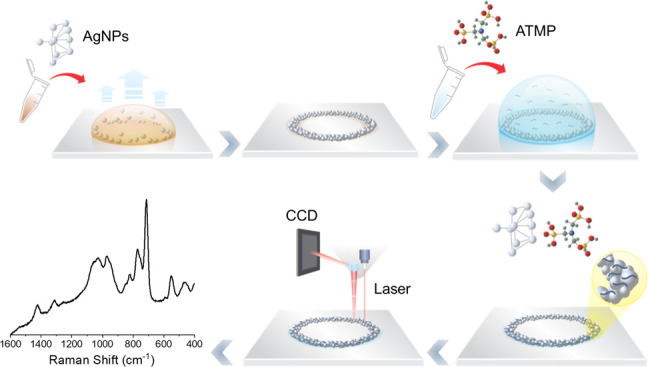

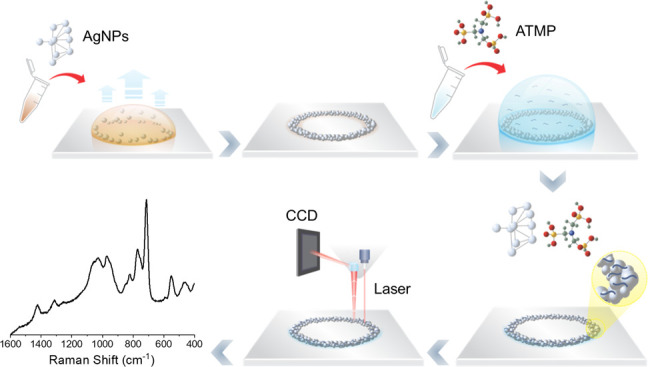

## 1 Introduction

Phosphonic acids (or phosphonates) are organophosphorus compounds characterized by the presence of one or more acidic functional groups, typically represented by the general formula R-PO­(OH)_2_.^1,2^ The central phosphorus atom is bonded to one doubly bonded oxygen atom (P=O), two hydroxyl groups (P–OH), and one organic group (R-P), creating a tetrahedral geometry.^3^ This structural configuration is reinforced by the covalent carbon–phosphorus bond, which contributes to the stability of the molecule.^4^ This unique arrangement of these functional groups contributes to several important physicochemical characteristics, including water solubility,^1,5^ chemical stability,^6^ ability to form chelates with metal ions,^7–9^ resistance to corrosion/oxidation,^10,11^ adsorption on various mineral surfaces,^12^ and supramolecular properties.^1^ These characteristics make phosphonates effective in various applications such as scale inhibition,^11,13–15^ corrosion control,^16–18^ water treatment,^5,19,20^ minerals processing,^21,22^ cancer treatment,^23^ and so on.

Phosphonic acids can be categorized into two main groups: nitrogen-free phosphonates and aminophosphonates.^5^ Nitrogen-free phosphonates may also contain carboxyl and hydroxyl groups such as 1-hydroxyethane-1,1-diphosphonic acid (HEDP) and 2-hydroxyphosphonoacetic acid (HPAA).^24^ On the other hand, aminophosphonates have a structure that includes the amino functional group and three to five phosphonate groups (polyphosphonates), making them highly effective in binding metal ions. The compounds belonging to this latter group are generally produced by first synthesizing phosphorous acid through the reaction of PCl_3_ with water.^6^ The resulting acid is then combined with formaldehyde and reacts with either ammonia to form amino tris­(methylenephosphonic acid) (ATMP) or various amines to produce ethylenediamine tetra­(methylenephosphonic acid) (EDTMP), hexamethylenediamine tetra­(methylenephosphonic acid) (HDTMP), or diethylenetriamine penta­(methylenephosphonic acid) (DTPMP), depending on the specific compound required.

ATMP and DTPMP are aminophosphonates that have been extensively studied and utilized in a wide range of applications. ATMP consists of a single central nitrogen atom bonded to three methylenephosphonic acid groups (Figure 1a). DTPMP, on the other hand, has a diethylenetriamine backbone, with each nitrogen atom bonded to one or more methylenephosphonic acid groups (Figure 1b). In total, it contains five methylenephosphonic acid groups.

**Figure 1:**
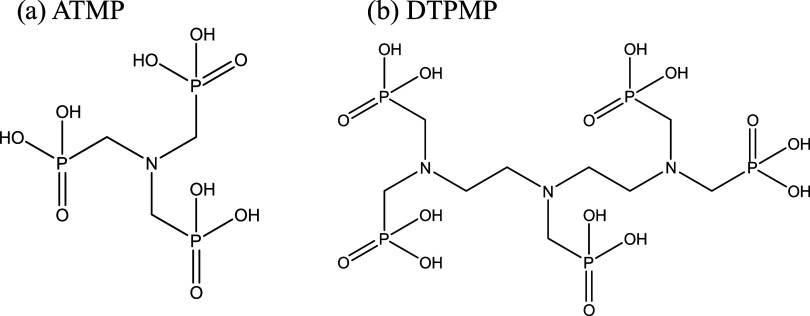
(a) ATMP and (b) DTPMP chemical structures.

The use of both molecules in various industrial applications is associated with the presence of a tetracoordinated phosphorus atom bonded to oxygen atoms, which allows these phosphonates to effectively chelate with a variety of metal ions, such as Ca^2+^, Mg^2+^, and Zn^2+^, forming stable complexes.^12,25^ This property makes them effective as scale inhibitors, preventing the formation of mineral deposits, such as CaCO_3_ and CaSO_4_, in water and wastewater treatment systems.^26–28^ This mechanism inhibits the growth of crystal structures, while also promoting the dispersion of smaller particles into the surrounding solution.^29^ Additionally, ATMP and DTPMP are widely utilized to protect metal surfaces from corrosion in industrial environments, such as the oil and gas industry and water treatment facilities.^30,31^ In these systems, inhibitors adsorb onto the metal surface, creating a protective barrier that prevents interaction with oxidizing agents, thereby reducing the corrosion rate.^32^ Their chelating ability also contributes to their incorporation in cleaning products and detergents, where they aid in the removal of mineral deposits, thus enhancing cleaning effectiveness.^6,33^ In the paper and pulp industry, these phosphonates are employed to control mineral deposition during production, improving both product quality and process efficiency.^34^ Furthermore, ATMP and DTPMP are also used as stabilizers for metallic nanoparticles, such as iron nanoparticles, preventing agglomeration and oxidation.^35^ This stabilization is important in applications such as catalysis,^36^ environmental remediation,^37^ and water treatment,^38^ where maintaining the dispersion and reactivity of nanoparticles is essential for performance and efficiency.

Due to their various uses in industry, the consumption of organic phosphonates has increased greatly in recent decades. This is concerning since these compounds are often discharged into aquatic environments.^39,40^ Phosphonates degrade slowly, releasing bioavailable phosphate, which promotes algae growth and contributes to water eutrophication.^40^ Another issue with the accumulation of phosphonates is that their degradation can produce toxic byproducts.^41^ Additionally, their strong metal-binding properties enhance the mobility of heavy metals in water, thereby increasing environmental risks.^4^ Thus, the detection and control of phosphonate levels in aquatic environments have become a recurring concern.

In the literature, the main methodologies used for the detection of ATMP and DTPMP primarily rely on advanced chromatographic techniques. These include ion chromatography (IC),^42–44^ high-performance liquid chromatography (HPLC),^45^ HPLC with pulsed amperometric detection (HPLC-PAD),^46^ liquid chromatography coupled with mass spectrometry using a particle beam interface (LC/PB-MS),^47^ and ion chromatography combined with inductively coupled plasma mass spectrometry (IC-ICP-MS).^48^ However, these methods involve complex and time-consuming steps for sample preparation. Consequently, one of the challenges for the scientific and industrial community is the need to develop analytical methods for monitoring phosphonates discharged by industries in aquatic environments, such as ATMP and DTPMP, that offer operational simplicity, quick response times, and high sensitivity.^49^ In this way, researchers have been focusing on utilizing surface-enhanced Raman spectroscopy (SERS) for detection of a wide range of phosphonate compounds.^50–54^

This technique allows the detection of molecules when they are adsorbed or near metallic nanoparticles, such as silver and gold.^55^ The plasmonic properties of these metallic nanostructures enhance the Raman signal of the molecules, allowing for direct and rapid detection, even at low concentrations.^56,57^ In the literature, several articles are using SERS for the detection of different molecules and even microplastics at low concentration.^58–62^ However, the vibrational characterization and assignment of the vibrational modes of ATMP and DTPMP using Raman spectroscopy and SERS have not yet been documented in the literature.

This gap highlights the need for comprehensive studies to understand the vibrational modes of these phosphonates, which are essential for their effective monitoring and detection by SERS. Therefore, this study aims to investigate the theoretical vibrational spectra of ATMP and DTPMP using density functional theory (DFT). Additionally, experimental Raman and SERS spectra of these molecules were also obtained.

## 2 Materials and Methods

### 2.1 Materials

The materials employed in this investigation were aminotrimethylene phosphonic acid (ATMP) (Sigma, 97.0%), diethylenetriamine penta­(methylenephosphonic acid) (DTPMP) (Sigma, 50.0%), silver nitrate (AgNO_3_) (Synth, 99.0%), and sodium borohydrate (NaBH_4_) (Sigma 99.0%). All reagents were utilized without undergoing purification. Ultrapure water (Milli-Q) with an average resistivity of 18.25 MΩ cm^–1^ was employed in all experiments.

### 2.2 Equipment

The optical absorption UV–vis analysis of AgNPs was conducted by using an Agilent CARY 7000 spectrometer across the spectral range from 200 to 800 nm. Zeta potential measurements were obtained by using dynamic light scattering from a laser source at 633 nm with a Zetasizer Nano ZS instrument. Transmission Electron Microscopy (TEM) measurements of AgNPs were performed utilizing a Jeol model 2100 PLUS microscope operating at 200 kV voltage. Confocal Microscopy measurements were carried out using an Olympus model LEXT OLS4000 confocal microscope. The AgNPs colloid was centrifuged using a Thermo Scientific Heraeus Megafuge 8 centrifuge. Raman and SERS analyses were recorded using a Renishaw inVia Raman spectrometer with a semiconductor laser at 785 nm, equipped with a microscope using a 50× (NA = 0.50) objective lens. The Raman spectrum of the ATMP and DTPMP solutions was also obtained by using a Bruker Vertex70-RAMII FT-Raman spectrometer, equipped with a 1064 nm laser.

### 2.3 Computational Details

Quantum mechanical calculations were performed using Gaussian 09 and Gauss View 5.0 packages (Walingford).^63^ The DFT calculations were employed utilizing the BPV86 functional (Burke and Perdew’s 1986 functional with correlation replaced by Vosko et al.).^64,65^ The BPV86 functional was chosen because it performed better in the previous experiments than did the B3LYP and PBE functionals. The triple-ζ 6–311+G­(2dp) basis set was used for all atoms (carbon, hydrogen, oxygen, nitrogen, and phosphorus) except for the silver, which was simulated by using LANL2DZ effective core potential, to describe the inner shell and valence electrons of silver atoms. In the calculations, the Self-consistent Reaction Field (SCRF) Integral Equation Formalism Continuum Polarizable Model (IEFPCM) method was used to simulate solvation (water). Cartesian coordinates and geometrical parameters of all optimized structures are presented in Tables S1–S4 and S7–S10 in the Supporting Information (SI). The theoretical Raman spectra generated through computational calculations are presented in terms of Raman activity (*A*_i_).^66^ Therefore, in this study, the *A*_i_ was converted into Raman intensity (*I*_i_) using the procedure described in eq 1.

1 $$I_{i}=\frac{\alpha A_{i}{\left(\nu_{0}-\nu_{i}\right)}^{4}}{\nu_{i}\left({1-e}^{-\mathrm{hc}\nu_{i}/k_{B}T}\right)}$$

In this equation, the factor α, equal to 10^–12^, is used to normalize the intensities of the bands. ν_0_ represents the excitation frequency (cm^–1^), while ν_i_ corresponds to the vibrational frequency (cm^–1^). *T* denotes the absolute temperature, and *h*, *c*, and *k*_B_ are the Planck and Boltzmann constants, respectively. The identification of vibrational modes was carried out using the VEDA 4xx software (Vibrational Energy Distribution Analysis).^67^

### 2.4 Silver Nanoparticles (AgNPs) Synthesis

The synthesis of AgNPs was conducted following a procedure utilizing NaBH_4_ as a reducing agent, adapted from the method described by Emonds and colleagues,^51^ which employs the modified Creighton method.^68^ In summary, 150 mL of 2.0 mM NaBH_4_ was placed in an ice bath (∼2 °C) and stirred continuously. Subsequently, 50 mL of 2.5 mM AgNO_3_ was added dropwise to the NaBH_4_ solution over a period of 3 to 5 min, changing from colorless to golden-yellow. Then, the suspension was stirred for at least 30 min and allowed to rest to reach equilibrium, and the final suspension presented a golden-brown color (Figure S1 from SI file).

### 2.5 Raman and SERS Measurements

For the Raman measurements of the solid and solution (100,000 ppm/3.35 × 10^–1^ mol L^–1^) of ATMP and acid solution of DTPMP (500,000 ppm/8.72 × 10^–1^ mol L^–1^), laser lines of 1064 and 785 nm were used. The 1064 nm laser on the FT-Raman measurements was operated at a power of 600 mW on the solution and 300 mW on the solid with 200 scans, while the 785 nm laser on the micro-Raman measurements was operated at a power of 11 mW with an acquisition time of 50 s.

SERS measurements were conducted employing the “coffee ring” effect.^62^ Initially, 1 mL of silver colloid underwent centrifugation at 3500g for 30 min. Subsequently, the supernatant was discarded, leaving approximately 100 μL of decanted colloid (Figure S1 from SI file). Following this, 5 μL of the concentrated AgNPs suspension was carefully dripped onto a silicon wafer, which was then dried for at least 1 h at room temperature. Then, 5 μL of the analytes ATMP and DTPMP, at different concentrations of 1000 ppm/3.35 × 10^–3^ mol L^–1^ to 10 ppm/3.35 × 10^–5^ mol L^–1^ and 1.74 × 10^–3^ to 1.74 × 10^–5^ mol L^–1^, respectively, were deposited onto the SERS substrate and allowed to dry for 1 h at room temperature. A blank experiment was performed; in this case, 5 μL of H_2_O was deposited onto the SERS substrate instead of ATMP/DTPMP solutions. Measurements were conducted on three different points at the edges of the resulting AgNPs “coffee ring” (Figure S2 from the SI file). The laser (785 nm) power was kept at 300 μW with an acquisition time of 90 s.

## 3 Results and Discussion

### 3.1 Raman Spectroscopy of ATMP and DTPMP and Its DFT Modeling

Initially, the optimized ATMP and DTPMP molecular geometries were obtained by using the Gaussian 09 software package to determine the most stable conformations of these phosphonate molecules. Figure S3 illustrates the optimized structures of both molecules, showing their spatial arrangements and main structural features. Afterward, the vibrational frequencies of these molecules were calculated. The following text presents a discussion of the vibrational modes calculated by DFT, making a comparison between the theoretical and experimental Raman spectra. Additionally, the assignments carried out using the VEDA software for both molecules are presented. Furthermore, a comparative analysis of the vibrational assignments made in studies of literature of organic molecules containing phosphonate groups is provided.

Experimental Raman analysis of ATMP was performed on both the solid-state and its aqueous solution (100,000 ppm/3.35 × 10^–1^ mol L^–1^), with pH ∼2, using the laser source at 1064 and 785 nm. Figure 2 presents the theoretical and experimental spectra of ATMP (λ_0_ = 1064 nm), covering the range from 3200 to 400 cm^–1^. In the theoretical spectrum (Figure 2A), bands are observed above 2900 cm^–1^ and within the range of 1500 to 400 cm^–1^. This spectrum is similar to the experimental spectra (Figure 2B,C), exhibiting bands in the same regions. Through a comparison of the experimental Raman spectra of the ATMP solid (Figure 2B) and solution (Figure 2C), it is observed that the latter spectrum exhibits band broadening compared to the former. This is due to the interaction between ATMP molecules and water. This interaction creates a hydrogen-bonding network and induces solvation effects, leading to variations in vibrational energy levels and, consequently, broader spectral features.^52^

**Figure 2:**
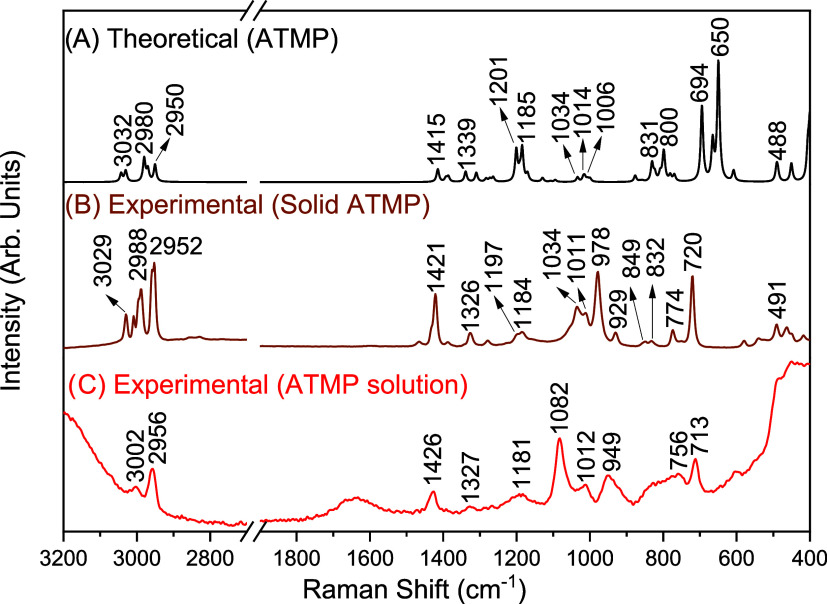
(A) Theoretical and experimental Raman spectra (λ_0_ = 1064 nm) of ATMP in both (B) solid-state and (C) solution (100,000 ppm/3.35 × 10^–1^ mol L^–1^).

In the high-wavenumber region of the experimental solid-state Raman spectrum of ATMP, bands appear at 3029, 3008, 2994, 2988, and 2952 cm^–1^, corresponding to CH stretching vibrations characteristic of organic compounds.^69,70^ In the solution spectrum, only two bands, assigned to this vibrational mode, are observed at 3002 and 2956 cm^–1^. The bands related to the deformation of the CH_2_ group are located at 1421 and 1426 cm^–1^ in the Raman spectra of the solid and solution phases of ATMP, respectively. This assignment aligns with reports in the literature where similar vibrational modes are identified in various organic compounds.^69–72^ The bands at 1326 cm^–1^ in the solid phase and at 1327 cm^–1^ in the solution correspond to ν­(NC), δ­(HCN), and τ­(HCNC) vibrations. In the literature, some studies associate bands in this region with vibrations occurring between the N and C atoms of phosphonomethyl groups bonded to amino groups. Podstawka and collaborators observed bands in this region assigned to NC stretching in phosphonate derivatives of imidazole, thiazole, and pyridine.^73^ The deformation of the CNC group was observed by Mikac and collaborators in glyphosate molecules at 1342 cm^–1^.^74^ In another study, Podstawka and collaborators also assigned bands around 1340 cm^–1^ to (NC­(H,C)­C) and NCH_2_C bending in phosphonate tripeptides.^53^

In the region below 1300 cm^–1^, the Raman spectrum of ATMP is primarily characterized by vibrations associated with the phosphonate group. The P=O stretching mode, when OH groups are bonded to the phosphorus atom, typically appears as a medium-intensity band in Raman spectra.^69^ A variety of studies available in the literature that assign Raman bands for molecules containing phosphonate groups indicates that the bands associated with this vibration are predominantly located in the range of 1148–1280 cm^–1^.^53,70,73,75^ In the theoretical Raman spectrum of ATMP (Figure 2A), two bands at 1201 and 1185 cm^–1^ are assigned to this stretching vibration. These bands are observed as a shoulder at 1197 cm^–1^ and a band at 1185 cm^–1^ in the Raman spectra of the solid phase, respectively, and as a broadened band centered at 1181 cm^–1^ in the spectrum of the ATMP solution. The bands at 1034, 1011, and 978 cm^–1^ in the Raman spectrum of solid ATMP are associated with deformation of the HOP group. These bands broaden and shift to 1082, 1012, and 949 cm^–1^, respectively, in the Raman spectrum of the ATMP solution. In the literature, it is reported that the bands present in the region between 1060 and 925 cm^–1^ are assigned to the stretching of the P–O bond.^70,76–78^

In the Raman spectrum of solid ATMP, two weak bands are also observed at 849 and 832 cm^–1^. In the solution spectrum, these bands are observed as a broad shoulder of the 756 cm^–1^ band. In the theoretical spectrum of ATMP, two bands are identified in this region at 831 and 800 cm^–1^, corresponding to ν­(NC) + ν­(PO) and ν­(PO) + ν­(PC), respectively. Holanda and collaborators attribute the band around 853 cm^–1^ to PO stretching and the band at approximately 818 cm^–1^ to PO and PC stretching in the glyphosate molecule.^79^ Piergies and collaborators reported a band at 825 cm^–1^, assigned to PO stretching in the N-benzylamino-(4-boronphenyl)-R-methylphosphonic acid molecule.^80^ Podstawka and collaborators assigned a band in this region for phosphonodipeptides to ν­(OPO).^81^

According to Costa and collaborators, two bands, approximately at 780 and 720 cm^–1^, are associated with ν­(P–OH) and ν­(PC) vibrations in the spectrum of the glyphosate molecule.^82^ Feis and co-workers assigned these bands to ν­(C–P) + ν­(C–P–OH) and ν­(C–P) + ν­(P–OH) + ν­(C–P–OH), respectively, for the same molecule.^54^ These bands are also observed in the solid-state spectrum, at 774 and 720 cm^–1^, and in the solution spectrum, at 756 and 713 cm^–1^. According to DFT calculations and the assignment made using the VEDA software, these modes are associated with ν­(PC) + δ­(PCN) and ν­(PO) + ν­(PC) + δ­(PCN), respectively. Moreover, a band associated with the deformation of the OPO and CNC modes is observed at 491 cm^–1^ in the solid ATMP Raman spectrum. In the solution spectrum, this band shifts to 484 cm^–1^. In the literature, the deformation of the OPO group for the glyphosate molecule is observed at approximately 454 cm^–1^.^79^

The Raman spectra of solid and solution-phase ATMP using the 785 nm laser source are presented in Figure S4 in the SI. Similar bands to those observed with the 1064 nm laser line are also detected in these spectra. Table 1 presents the wavenumbers (for both the 1064 and 785 nm laser lines) and the vibrational assignments for the most significant Raman bands of ATMP, comparing theoretical predictions with experimental data. Additionally, Table S5 in the SI shows the complete assignments of all calculated Raman vibrational modes.

**Table 1:** Theoretical (BPV86/6–311 + G­(2d,p)) and Experimental (Solid-State and Solution 100,000 ppm/3.35 × 10^–1^ mol L^–1^, λ_0_ = 1064 and 785 nm) Wavenumbers of ATMP with the Respective Assignments Based on the Potential Energy Distribution Computed with the VEDA 4xx Software^a^

theoretical ATMP Raman wavenumbers (cm^–1^) (DFT/BPV86)	experimental solid ATMP Raman wavenumbers (cm^–1^) (λ_0_ = 1064 nm)	experimental solid ATMP Raman wavenumbers (cm^–1^) (λ_0_ = 785 nm)	experimental ATMP solution Raman wavenumbers (cm^–1^) (λ_0_ = 1064 nm)	experimental ATMP solution Raman wavenumbers (cm^–1^) (λ_0_ = 785 nm)	assignments^b^
3043	3029 w	3037 vw			ν(C_3_H_5_) (88%)
3032	3008 w	3017 vw			ν(C_7_H_8_) (14%) + ν(C_10_H_11_) (19%) + ν(C_10_H_12_) (56%)
2980	2994 sh	2997 w	3002 w	2971 m	ν(C_7_H_8_) (37%) + ν(C_7_H_9_) (57%)
2970	2988 s	2966 w			ν(C_10_H_11_) (72%) + ν(C_10_H_12_) (23%)
2950	2952 vs	2959 w	2956 m		ν(C_3_H_4_) (90%)
1415	1421 s	1425 s	1426 w	1440 m	δ(H_4_C_3_H_5_) (34%) + δ(H_9_C_7_H_8_) (17%) + δ(H_12_C_10_H_11_) (24%)
1339	1326 w	1330 w	1327 vw	1342 m	ν(N_6_C_10_) (11%) + ν(N_6_C_3_) (10%) + δ(H_8_C_7_N_6_) (39%) + τ(H_9_C_7_N_6_C_10_) (10%)
1201	1196 sh	1200 sh	1213 sh	1208 w	ν(P_1_O_2_) (22%) + ν(P_13_O_14_) (35%) + δ(H_11_C_10_N_6_) (12%)
1185	1184 w	1185 vw	1181 vw		ν(P_15_O_16_) (81%)
1034	1034 m	1034 m	1082 s	1095 vs	δ(H_22_O_21_P_13_) (80%)
1014	1011 sh	1014 m	1012 w	1027 m	δ(H_28_O_27_P_15_) (80%)
1006	978 vs	982 s	949 m	958 s	δ(H_24_O_23_P_13_) (59%) + δ(H_26_O_25_P_15_) (23%)
831	849 vw	862 w			ν(N_6_C_7_) (11%) + ν(N_1_C_19_) (11%) + ν(P_1_O_17_) (20%)
800	832 vw	851 sh		849 w	ν(P_15_O_27_) (54%) + ν(P_15_C_7_) (12%)
694	774 w	775 w	756 w	765 m	ν(P_1_C_3_) (44%) + δ(P_1_C_3_N_6_) (10%)
664		754 vw			ν(O_21_H_22_) (97%)
650	720 vs	721 vs	713 m	725 s	ν(P_15_O_27_) (15%) + ν(P_15_C_7_) (48%) + δ(P_15_C_7_N_6_) (14%)
400	491 w	491 m	484 sh	460 m	δ(O_21_P_13_O_14_) (19%) + δ(C_7_N_6_C_3_) (21%)

a ν, stretching; δ, in-plane deformation, τ, torsional; vs, very strong; s, strong; m, medium; vw, very weak; w, weak; sh, shoulder.

b All assignments include internal coordinates that contribute 10% or more to the PED.

The DTPMP reagent is supplied only as a solution containing 50% active compound in a mixture of hydrochloric acid (HCl) and water. The Raman analyses were performed using a solution with a concentration of 500,000 ppm/8.72 × 10^–1^ mol L^–1^ (pH ∼2). The theoretical and experimental spectra using the laser source at 1064 nm of DTPMP in the range of 3200 to 400 cm^–1^ are presented in Figure 3.

**Figure 3:**
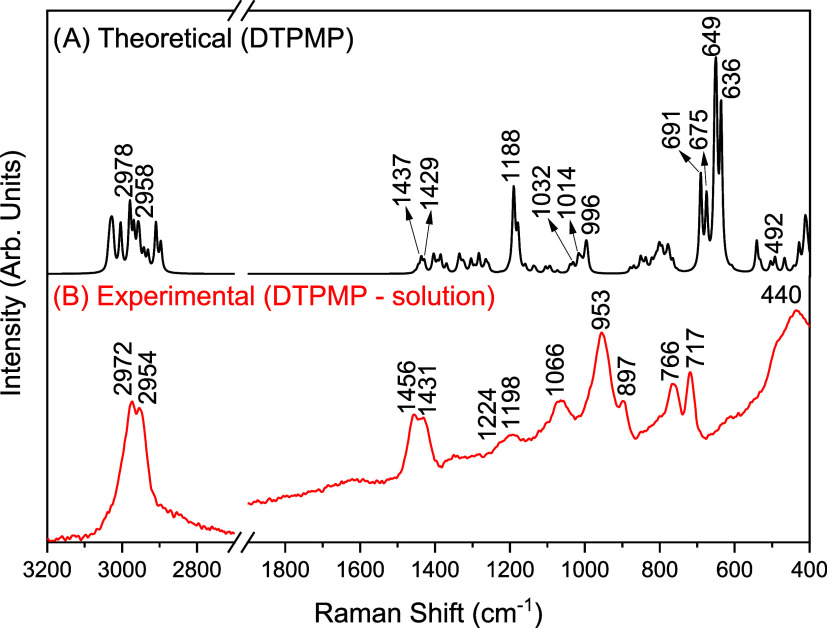
(A) Theoretical and (B) experimental Raman spectra (λ_0_ = 1064 nm) of DTPMP solution (500,000 ppm/8.72 × 10^–1^ mol L^–1^) in the range of 3200 to 400 cm^–1^.

In the theoretical spectrum of DTPMP (Figure 3A), some bands are observed above 2900 cm^–1^, as discussed earlier, these bands are common in organic compounds and are assigned to the CH stretching.^69,83,84^ In the experimental spectrum (Figure 3B), these bands are observed at 2972 and 2954 cm^–1^. Two bands at 1456 and 1431 cm^–1^ in the experimental spectrum are assigned to the deformation of CH_2_.^69,71,72^ In the range between 1400 and 1100 cm^–1^, a low-intensity band at 1198 cm^–1^ with a shoulder at 1224 cm^–1^ are observed. This band corresponds to the theoretical bands at 1179 and 1188 cm^–1^, respectively, which are assigned to the ν­(P=O) and δ­(HOP) vibrations.

The band at 1066 cm^–1^ in the Raman spectrum of the solution is associated with δ­(HOP), as observed for the ATMP molecule. In addition, this band also includes the contribution of C–C stretching vibrations due to the presence of ethylene groups, which are absent in the ATMP structure. According to Lin-Vien and co-workers, this vibrational mode can be observed in the range of 1132 to 885 cm^–1^.^69^ The bands at 1014, 996, and 897 cm^–1^ in the theoretical spectrum appear overlapped at 953 cm^–1^ in the experimental spectrum, assigned to the deformation of the HOP group. The bands at 691 and 675 cm^–1^ in the theoretical spectrum are attributed to ν­(PO), ν­(PC), and δ­(PCN) and also appear overlapped at 766 cm^–1^ in the experimental spectrum. The same applies to the theoretical bands at 649 and 636 cm^–1^, which are observed at 717 cm^–1^ in the experimental spectrum. These bands are associated with ν­(PC) + δ­(PCN) and ν­(PC) + δ­(PCN) + ν­(PO) vibrations, respectively. As observed in the Raman spectrum of ATMP, the δ­(OPO) vibrational mode appears at 440 cm^–1^ in the experimental spectrum.

Using the 785 nm laser source, the Raman spectrum of the DTPMP solution (Figure S5 from SI file) shows bands at 2950, 1430, 1053, 951, 763, and 718 cm^–1^ on a fluorescence background. These bands correspond to those at 2954, 1431, 1066, 953, 766, and 717 cm^–1^ in the spectrum obtained using the 1064 nm laser, respectively.

Table 2 presents the vibrational assignments for the main theoretical and experimental Raman bands of DTPMP using laser sources at both 1064 and 785 nm. The complete assignments of all calculated vibrational modes are shown in Table S6 in the SI. Theoretical calculations and experimental analyses for obtaining the IR spectra for both molecules, ATMP and DTPMP, were also performed. The spectra are shown in Figure S6 in the SI. The assignments for the IR bands are presented in Tables S5 and S6 for ATMP and DTPMP, respectively.

**Table 2:** Theoretical (BPV86/6–311 + G­(2d,p)) and Experimental (Solution 500,000 ppm/8.72 × 10^–1^ mol L^–1^), Raman (λ_0_ = 1064 and 785 nm) Bands of DTPMP with the Respective Assignments Based on the Potential Energy Distribution Computed with the VEDA 4xx Software^a^

theoretical DTPMP Raman wavenumbers (cm^–1^) (DFT/BPV86)	experimental DTPMP solution Raman wavenumbers (cm^–1^) (λ_0_ = 1064 nm)	experimental DTPMP solution Raman wavenumbers (cm^–1^) (λ_0_ = 785 nm)	assignments^b^
2978	2972 vs		ν(C_34_H_35_) (61%) + ν(C_34_H_36_) (37%)
2958	2954 sh	2950 vw	ν(C_30_H_31_) (72%) + ν(C_30_H_32_) (24%)
1437	1456 s		δ(H_2_C_1_H_3_) (38%) + δ(H_25_C_23_H_24_) (21%) + δ(H_32_C_30_H_31_) (11%)
1429	1431 sh	1430 vw	δ(H_39_C_37_H_38_) (10%) + δ(H_29_C_27_H_28_) (13%) + δ(H_32_C_30_H_31_) (47%)
1188	1226 sh		ν(P_11_O_12_) (23%) + ν(P_53_O_46_) (20%) + ν(P_54_O_43_) (27%) + δ(H_56_O_55_P_54_) (10%)
1179	1198 vw		ν(P_13_O_14_) (74%)
1039			ν(C_1_C_23_) (12%)
1032	1066 m	1053 m	ν(C_1_C_23_) (39%) + δ(H_16_O_15_P_11_) (21%)
1014			δ(H_18_O_17_P_11_) (80%)
996	953 s	951 s	δ(H_51_O_50_P_52_) (55%) + δ(H_60_O_59_P_52_) (19%)
691	766 m	763 m	ν(P_53_O_57_) (15%) + ν(P_53_C_57_) (45%) + δ(P_53_C_37_N_33_) (12%)
675			ν(P_11_C_8_) (39%) + δ(P_11_C_8_N_4_) (11%) + ν(P_11_O_17_) (12%) + ν(P_11_O_15_) (17%)
649	717 m	718 s	ν(P_54_C_40_) (52%) + δ(P_54_C_40_N_33_) (16%)
636			ν(P_13_C_5_) (47%) + δ(P_13_C_5_N_4_) (15%) + ν(P_13_O_21_) (13%)
411	440 s		δ(O_43_P_54_O_55_) (16%)

a ν, stretching; δ, in-plane deformation; vs, very strong; s, strong; m, medium; vw, very weak; w, weak; sh, shoulder.

b All assignments include internal coordinates that contribute 10% or more to the PED.

### 3.2 SERS Spectroscopy of ATMP and DTPMP and Its DFT Modeling

#### 3.2.1 Characterization of AgNPs

Figure 4 shows the UV–vis absorption spectra of the colloidal suspension of NaBH_4_ reduced-AgNPs on the day of synthesis and over the following 120 days (stored under refrigeration at 4–8 °C). The spectra exhibit very similar profiles, with the maximum of LSPR band centered around 389 nm, which is expected for the nanostructure synthesized by the proposed method of Emonds-alt and co-workers.^51^

**Figure 4:**
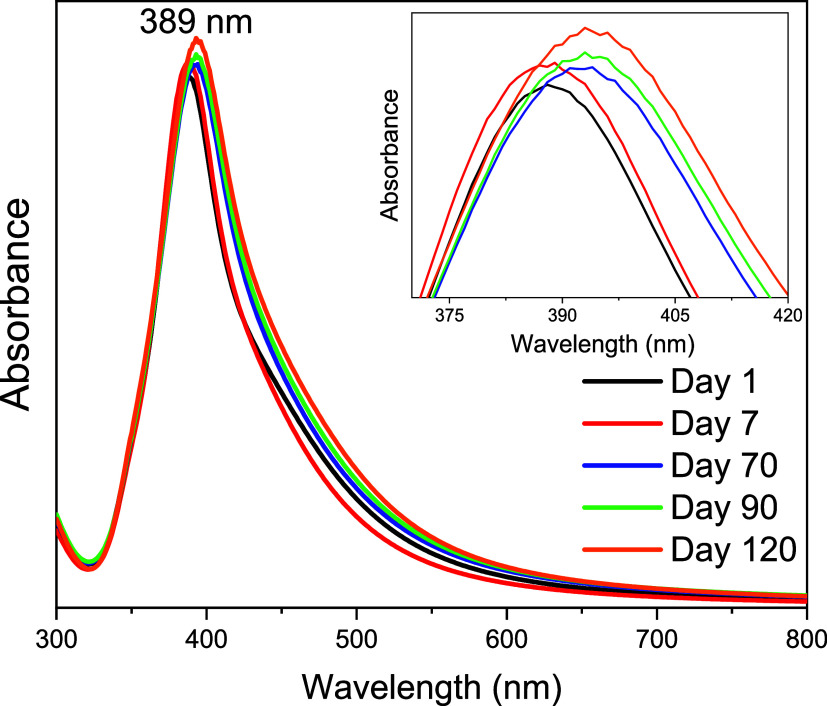
UV–vis optical absorption spectra of NaBH_4_ reduced-AgNPs over 120 days. Insert: Zoom at maximum of the AgNPs LSPR band.

After 120 days, a red shift of the LSPR maximum to 393 nm was observed. This slight shift is expected, due to the fact that over time, the NaBH_4_ molecules in the suspension that stabilize the NPs degrade, leading to NP aggregation. However, this aggregation is slow enough during the observation period, considering the NPs suspensions are stable over this period.^85,86^ Another slight change in the spectral profile is the intensity increase. This increase is due to the excess of NaBH_4_, which causes the synthesis to continue very slowly, and, therefore, the formation of AgNPs continues gradually.^87^

The ζ potential measurements were also performed immediately after synthesis and over 120 days. The measured values fell within the range of −30 to −40 mV, suggesting high stability.^88,89^

Figure 5A–C shows the TEM micrographs of AgNPs, and the predominant presence of spherical or quasi-spherical nanoparticles. The histogram in Figure 5D shows a medium diameter of 27 ± 7 nm with diameters between 20 and 30 nm; these results corroborate with the UV–vis characterization.

**Figure 5:**
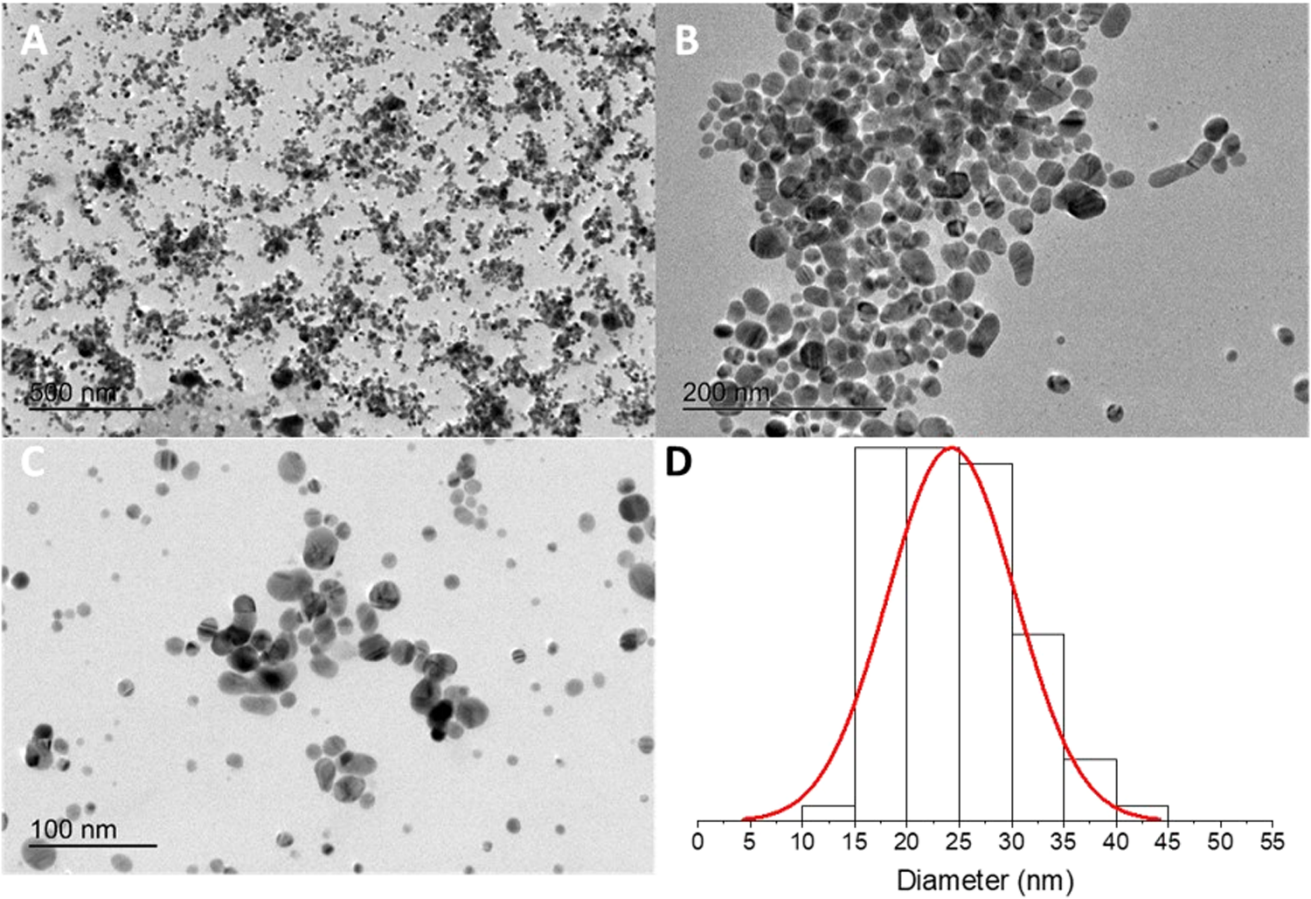
TEM micrographs of colloidal suspensions at different magnifications (A, B, and C). Histogram describing the diameter of the distribution of the nanoparticles (D).

#### 3.2.2 DFT Modeling

In the literature, studies that have obtained the SERS spectra of molecules containing phosphonate groups suggest that the interaction with plasmonic nanoparticles occurs predominantly through these functional groups.^51^ Thus, the SERS spectra of ATMP and DTPMP were calculated by using the DFT method, employing a model in which the molecules interact through their phosphonate groups with an Ag_10_ cluster. This approach was used to investigate the interaction mechanism between the analytes and AgNPs. Cartesian coordinates of all optimized structures are presented in Tables S7–S10. The optimized structures for both molecules, along with a model of adsorption involving the Ag_10_ cluster and phosphonate groups, are presented in Figure S7 in the SI.

Figure 6A presents a comparison between the theoretical and experimental SERS spectra of ATMP in the range of 1500 to 400 cm^–1^. The theoretical spectrum highlights the vibrational modes associated with the phosphonate group, which interacts with the silver cluster. In line with this, the experimental SERS spectrum reveals an enhancement of the vibrational modes corresponding to the phosphonate groups. However, in this latter spectrum, notable changes in the intensity and wavenumber of some bands are observed when compared to the Raman spectrum of the ATMP solution performed using the 785 nm laser source (Figure S4B). These changes are associated with the chemical interaction of the phosphonate groups with the NPsAg.

**Figure 6:**
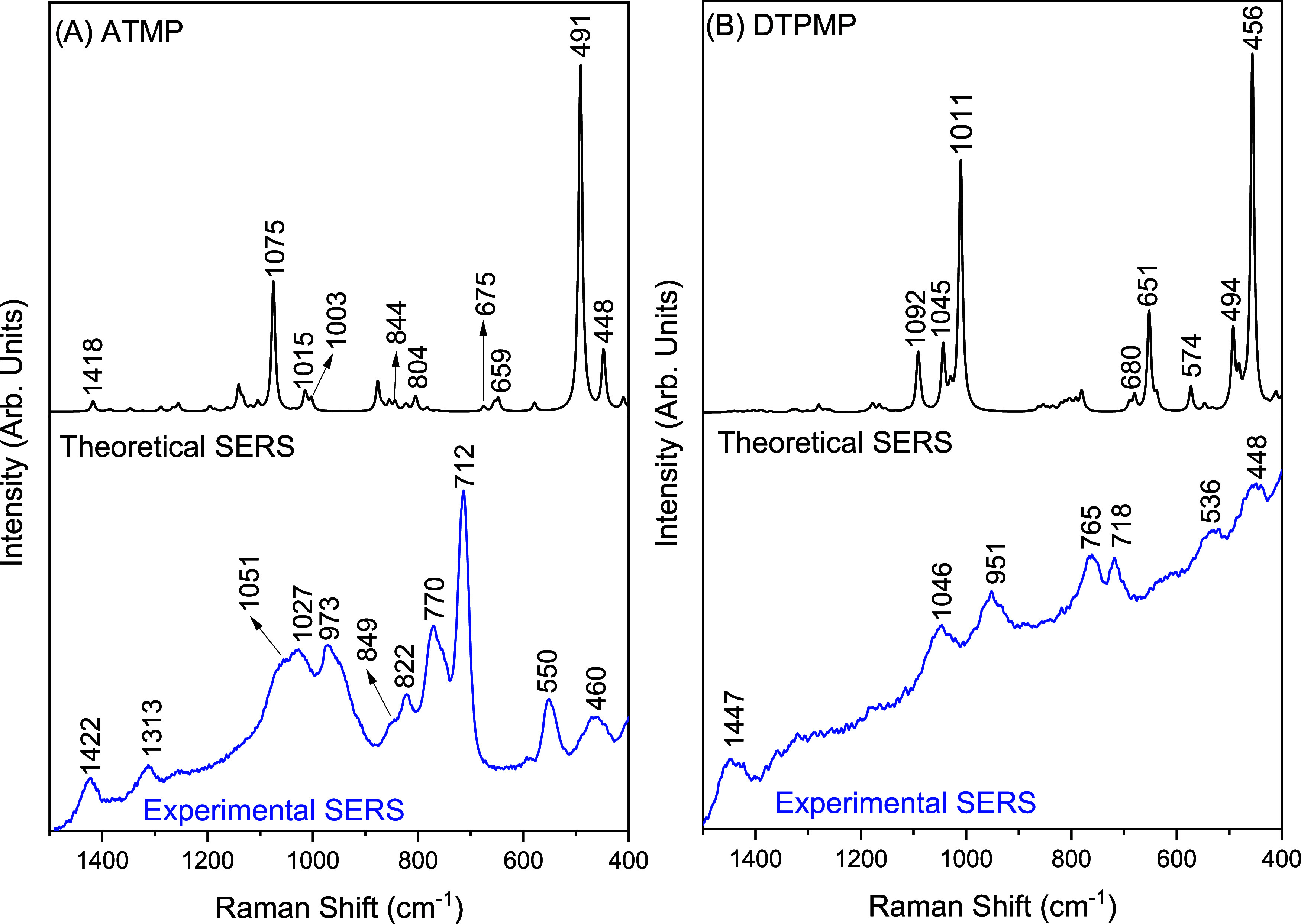
Theoretical and experimental SERS spectra of (A) ATMP (1000 ppm/3.34 × 10^–3^ mol L^–1^) and (B) DTPMP (1000 ppm/1.74 × 10^–3^ mol L^–1^) molecules.

The Raman bands of the ATMP solution at 1095, 958, 765, and 725 cm^–1^ shift to 1051, 973, 770, and 712 cm^–1^ in the SERS spectrum, respectively. These bands correspond to the vibrational modes δ­(HOP), δ­(HOP), ν­(PC) + δ­(PCN), and ν­(PO) + ν­(PC) + δ­(PCN), in that order. Notably, the bands at 973, 770, and 712 cm^–1^ become more intense, likely due to the proximity of the phosphonate groups with the surface of the NPsAg. The P=O stretching bands, typically observed between 1210 and 1180 cm^–1^, are absent in the SERS spectrum. This absence may be attributed to the horizontal orientation of the P=O bonds on the nanoparticle surface. According to the Moskovits selection rule, only vibrational modes perpendicular to the surface of plasmonic nanoparticles experience significant enhancement.^90^ The last bands observed in the experimental SERS spectrum at 550 and 460 cm^–1^ are associated with the torsional vibrational mode of the phosphonate group directly bound to the AgNPs (HOPAg). Table 3 presents the vibrational assignments for the main theoretical and experimental SERS bands of ATMP. Table S11 in the SI shows a complete assignment of all calculated SERS vibrational modes of ATMP.

**Table 3:** Theoretical and Experimental SERS Wavenumbers of the Main Bands of ATMP and Their Respective Assignments^a^

theoretical ATMP SERS wavenumbers (cm^–1^) (DFT/BPV86)	experimental ATMP SERS wavenumbers (cm^–1^) (λ_0_ = 785 nm)	assignments^b^
1418	1422 w	δ(H_4_C_3_H_5_) (19%) + δ(H_9_C_7_H_8_) (38%) + δ(H_12_C_10_H_11_) (15%)
1075	1051 sh	δ(H_28_O_27_P_15_) (25%) + δ(H_26_O_25_P_15_) (54%)
1015	1027 s	δ(H_28_O_27_P_15_) (48%) + δ(H_26_O_25_P_15_) (34%)
1003	973 s	δ(H_28_O_27_P_15_) (25%) + δ(H_26_O_25_P_15_) (54%)
844	849 sh	ν(N_6_C_3_) (10%) + ν(N_6_C_7_) (14%) + ν(N_6_C_10_) (11%) + ν(P_1_O_17_) (31%)
804	822 w	ν(N_6_C_3_) (10%) + ν(P_13_O_23_) (32%) + ν(P_15_O_25_) (16%)
675	770 s	ν(P_1_C_3_) (52%) + δ(P_1_C_3_N_6_) (13%)
659	712 vs	ν(P_13_O_21_) (24%) + ν(P_13_C_10_) (42%)
491	550 m	τ(H_18_O_17_P_1_Ag_32_) (78%)
448	460 w	τ(H_28_O_27_P_15_Ag_33_) (57%)

a ν, stretching; δ, in-plane deformation, τ, torsional; vs, very strong; s, strong; m, medium; w, weak; sh, shoulder.

b All assignments include internal coordinates that contribute 10% or more to the PED.

Theoretical and experimental SERS spectra of DTPMP are presented in Figure 6B. The experimental SERS spectrum of DTPMP shows an enhancement of modes associated with the phosphonate group, exhibiting similarity to those observed for ATMP (Figure 6A). The bands at 1046 and 951 cm^–1^ are assigned to the bending vibrations of the POH segment. The first band, as previously mentioned, was located at 1052 cm^–1^ in the Raman spectrum. This shift may be attributed to the interaction of DTPMP with AgNPs via its phosphonate groups. Additionally, the bands at 765 and 718 cm^–1^, assigned to ν­(PC) + δ­(PCN) and ν­(PO) + ν­(PC), respectively, are also enhanced in the SERS spectrum. As in the SERS ATMP spectra, the last bands observed in the experimental SERS spectrum at 536 and 448 cm^–1^ are associated with the phosphonate group’s torsional vibrational mode closer to the AgNPs cluster. Table 4 presents the vibrational assignments for the main theoretical and experimental SERS bands of DTPMP. Table S12 in the SI shows a complete representation of all calculated SERS vibrational modes of ATMP.

**Table 4:** Theoretical and Experimental SERS Wavenumbers of the Main Bands of DTPMP and Their Respective Assignments^a^

theoretical DTPMP SERS wavenumber (cm^–1^) (DFT/BPV86)	experimental DTPMP SERS (1000 ppm/1.74 × 10^–3^ mol L^–1^) wavenumber (cm^–1^) (λ_0_ = 785 nm)	assignments^b^
1441	1447 m	δ(H_2_C_1_H_3_) (31%) + δ(H_29_C_27_H_28_) (20%) + δ(H_32_C_30_H_31_) (20%)
1045	1046 m	δ(H_56_O_55_P_54_) (33%) + δ(P_11_O_15_H_16_) (17%) + δ(H_45_O_44_P_54_) (17%) +
1011	951 s	δ(H_45_O_44_P_54_) (58%) + δ(H_18_O_17_P_11_) (11%) + δ(H_56_O_55_P_54_) (17%)
680	765 s	ν(P_11_O_15_) (13%) + ν(P_11_O_17_) (16%) + ν(P_11_C_8_) (40%)
651	718 m	ν(P_52_C_34_) (43%) + δ(P_52_C_34_N_26_) (22%)
574	536 w	τ(H_56_O_55_P_54_C_40_) (69%)
456	448 m	δ(O_44_P_54_O_43_) (11%) + τ(H_45_O_44_P_54_C_40_) (61%)

a ν, stretching; δ, in-plane deformation; τ, torsional; s, strong; m, medium; w, weak.

b All assignments include internal coordinates that contribute 10% or more to the PED.

#### 3.2.3 SERS Detection of ATMP and DTPMP

AgNPs were applied as SERS substrates for ATMP and DTPMP detection in different concentrations of aqueous solutions by using the “coffee ring” effect. The concentration of AgNPs by centrifugation and the use of the “coffee ring” effect increase the number of hot spots and the controlled aggregation of the nanoparticles,^62,91^ allowing the use of the 785 nm laser, which was a better excitation for ATMP and DTPMP. Figure 7 presents the average of the three SERS spectra of ATMP at concentrations from 1000 to 10 ppm (3.35 × 10^–3^ to 3.35 × 10^–5^ mol L^–1^) obtained in the edges of the “coffee ring” border and the Raman spectrum of AgNPs (H_2_O). The most intense bands at 1051, 1027, 973, 770, and 712 cm^–1^ referring to the phosphonate group were used to monitor the variation of the SERS signal with the concentration of ATMP. It is possible to observe that these bands decrease in intensity when the concentration of ATMP is reduced. Until 50 ppm (1.67 × 10^–4^ mol L^–1^), it is possible to observe the phosphonate bands at 770 and 712 cm^–1^, indicating the detection of ATMP and the bands at 550 and 460 cm^–1^ of the bond between Ag and ATMP. The other bands suffer great interference at 50 ppm. At 10 ppm, the observed bands are similar to the bands observed in the spectrum of the blank experiment, where H_2_O was added under AgNPs instead of ATMP solutions, indicating that at 10 ppm, it is not possible to observe the characteristic SERS bands of ATMP.

**Figure 7:**
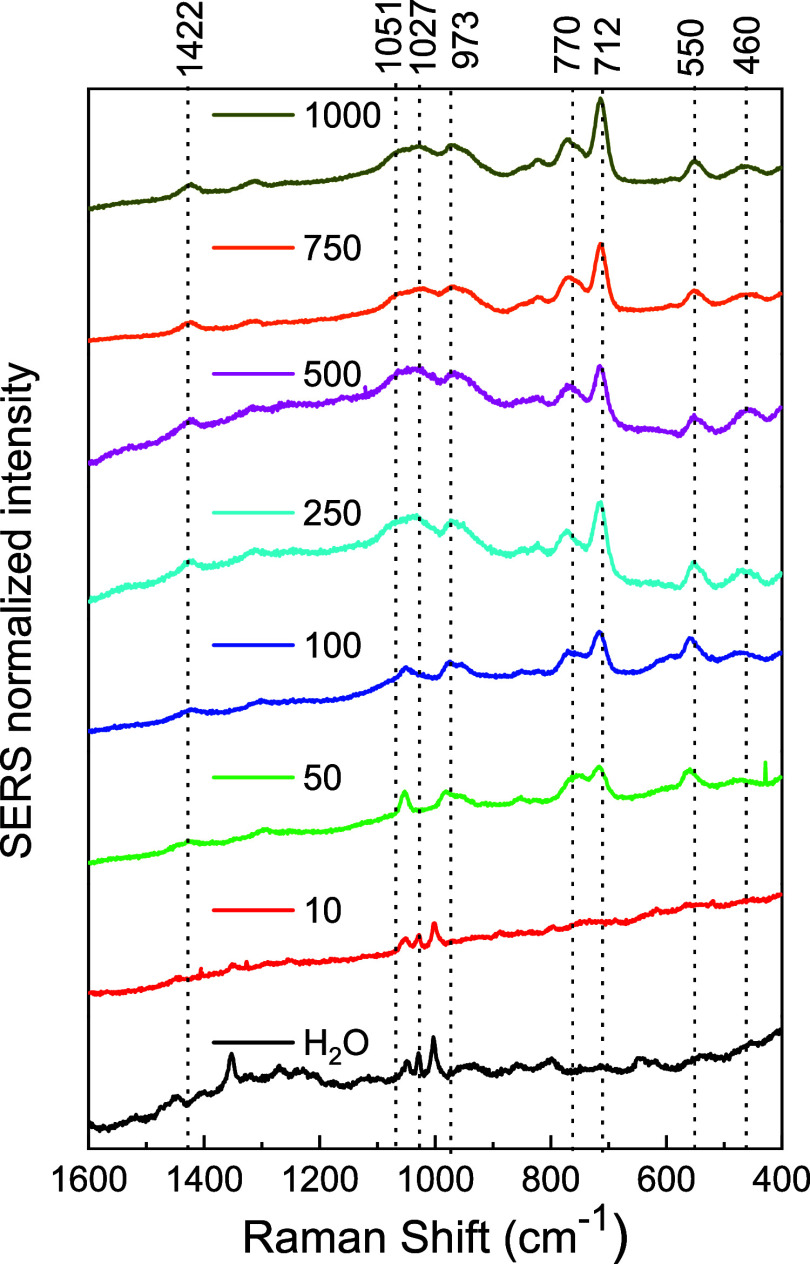
SERS spectrum of aqueous solution ATMP at different concentrations using the “coffee ring” effect excited at 785 nm (Range 1500–400 cm^–1^).

Figure 8 presents the average of the three SERS spectra of DTPMP at concentrations from 1000 to 10 ppm (1.74 × 10^–3^ to 1.74 × 10^–5^ mol L^–1^) obtained in the edges of the “coffee ring” border and the Raman spectrum of AgNPs (H_2_O). Analog to ATMP, the bands referring to the phosphonate group at 1046, 951, 765, and 718 cm^–1^ are the greater intensity in the SERS spectrum of DTPMP. These bands were used to monitor the variation of the SERS signal with the concentration of DTPMP. The intensity of the SERS signal of DTPMP diminished as its concentration was reduced. Up to 50 ppm (8.72 × 10^–5^ mol L^–1^), the detection of DTPMP was feasible through the observation of phosphonate bands at 765 and 718 cm^–1^, and the bands from the interaction between DTMP and AgNP cluster at 536 and 448 cm^–1^. Similar to the ATMP analysis, significant interference affects the other bands at 50 ppm. At a concentration of 10 ppm, the predominant spectral features were associated with the AgNPs. Still, different from ATMP, there is a weak signal of the bands at 1447, 765, and 448 cm^–1^, indicating the detection of DTPMP up to 10 ppm. Resembling the results of the blank experiment in which H_2_O was used instead of DTPMP solutions.

**Figure 8:**
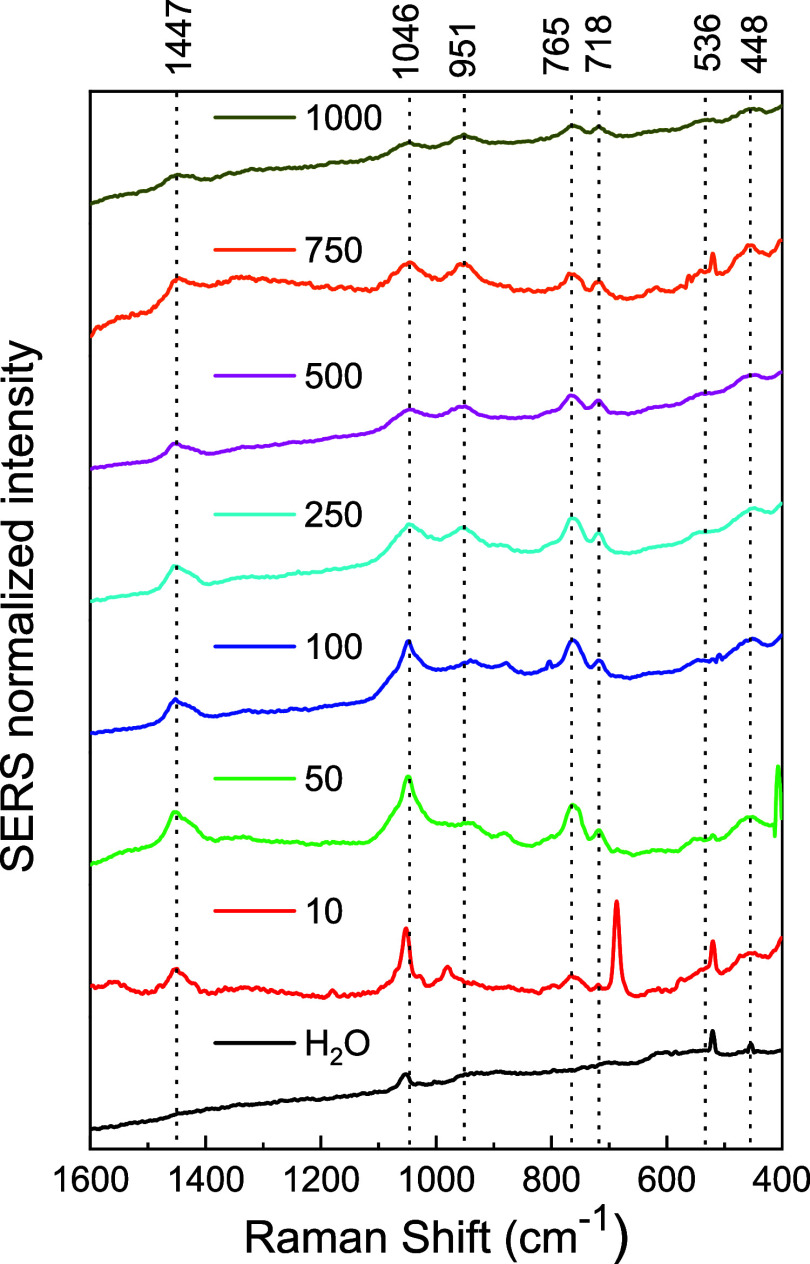
SERS spectrum of aqueous solution DTPMP at different concentrations using the “coffee ring” effect excited at 785 nm (Range 1600–400 cm^–1^).

## 4 Conclusions

This work presents a study of two widely used aminophosphonates, ATMP and DTPMP, with DFT analysis, Raman, SERS, and FT-IR, which are not observed in the literature. The results of the DFT analysis are consistent with the experimental results, and the vibrational mode assignments for both phosphonates are consistent with the similar molecules reported in the literature.

AgNPs reduced with NaBH_4_ were synthesized, and the method was adapted to Creighton synthesis, presenting good homogeneity in shape and size. This AgNPs were applied as the SERS substrate using the “coffee ring” effect with efficiency for both phosphonate molecules. DFT analysis from SERS using a Ag_10_ cluster showed the enhancement of some bands associated with the configuration of the molecule on the Ag surface. Both DFT calculations and experimental SERS spectra suggest that the molecules interact with the AgNPs from the phosphonate group. Using “coffee ring” method, it was possible to detect both molecules until 50 ppm and possible DTPMP until 10 ppm. This paper contributed to the literature with possible vibrational mode assignments to ATMP and DTPMP molecules, and the developed method is promising for an *in loco* detection of these molecules at low concentrations using SERS spectroscopy.

## Supplementary Material



## Notes

Table S1: the atomic coordinates for the optimized structure of ATMP; Table S2: geometrical parameters of ATMP; Table S3: the atomic coordinates for the optimized structure of DTPMP; Table S4: geometrical parameters of DTPMP; Figure S1: images of AgNPs colloid; Figure S2: confocal micrographs of AgNPs; Figure S3: optimized geometry by DFT of ATMP and DTPMP molecules; Figure S4: experimental Raman spectra of solid and solution phases of ATMP using 785 nm laser source; Table S5: complete theoretical and experimental (solid-state and solution, Raman and IR bands of ATMP, with the respective assignments); Figure S5: Raman spectrum of DTPMP solution using the laser source at 785 nm; Table S6: complete theoretical and experimental (solid-state and solution, Raman and IR bands of DTPMP, with the respective assignments); Figure S6: theoretical and experimental IR spectra of ATMP and DTPMP molecules.; Table S7: the atomic coordinates for the optimized structure of ATMP model adsorption of N–Ag10; Table S8: geometrical parameters of ATMP model adsorption of N–Ag10; Table S9: the atomic coordinates for the optimized structure of DTPMP model adsorption of N–Ag10; Table S10: geometrical parameters of DTPMP model adsorption of N–Ag10; Figure S7: DFT optimized structure of ATMP and DTPMP with a model of absorption Ag10-phosphonate group; Table S11: complete theoretical and experimental SERS wavenumbers of ATMP, with the respective assignments; Table S12: complete theoretical and experimental SERS wavenumbers of DTPMP with the respective assignments (PDF)
