# Supplementary material for: Investigation of Phosphonic Acids Based on Raman and Surface-Enhanced Raman Spectroscopy
Source: ACS Omega. 2025 Aug 27;10(35):40032–45. doi: 10.1021/acsomega.5c04862 (PMC12423975; doi:10.1021/acsomega.5c04862)
Supplement: Supplementary file 1 [file ao5c04862_si_001.pdf]

Supporting information of

Investigation of phosphonic acids based on Raman and surface-enhanced Raman spectroscopy

Linus Pauling de Faria Peixoto[a,c*], Bismark Nogueira da Silva[a], Regina Duque Estrada Carvalho[a], Rosane Alves Fontes[b], Luiz Alexandre Sacorague[b], Tiago Cavalcante Freitas[b], Jussara de Mello Silva[b], Giselle Maria Lopes Leite da Silva[b], Monica Teixeira da Silva[b], Cristiano Fantini[c] , Mariana Botelho Barbosa[a*], and Isabela Maria Ferreira Lopes[a].

[a] *Instituto SENAI de Inovação em Engenharia de Superfícies – Centro de inovação e Tecnologia CIT SENAI, Horto, Belo Horizonte, MG 31035-536, Brazil*

[b] *Centro de Pesquisas, Desenvolvimento e Inovação Leopoldo Américo Miguez de Mello – Cenpes/Petrobras, Ilha do Fundão, Rio de Janeiro, RJ 21941-915, Brazil*

[c] *Departamento de Física, Universidade Federal de Minas Gerais, Belo Horizonte, MG 31270-901 Brazil*

Table S1. The atomic coordinates for the optimized structure of ATMP.

Number	Element	X	Y	Z
**1**	P	-1.5452680	2.1325530	-0.1565580
**2**	O	-1.1319670	2.3741500	-1.7000810
**3**	C	-0.2262190	1.3028200	0.9452040
**4**	H	0.6263140	1.9988890	1.0169320
**5**	H	-0.6766130	1.1843520	1.9400920
**6**	N	0.2015250	0.0219250	0.4070080
**7**	C	1.1160970	0.0177350	-0.7098450
**8**	H	1.0214210	-0.9124910	-1.2920510
**9**	H	0.9303090	0.8788160	-1.3703180
**10**	C	-0.1199370	-1.2065150	1.0676710
**11**	H	0.7714650	-1.8451150	1.1965050
**12**	H	-0.5861040	-1.0291720	2.0460840
**13**	P	-1.3514480	-2.3713640	0.1116790
**14**	O	-1.9712410	-3.6917780	0.7899960
**15**	P	2.9581520	0.1260860	-0.1774440
**16**	O	4.1606580	0.1386550	-1.2508450
**17**	O	-1.8245360	3.5755600	0.7825450
**18**	H	-2.4122030	4.2348270	0.3311620
**19**	O	-3.0240270	1.2680710	0.0788700
**20**	H	-2.9149500	0.2638180	-0.1265070
**21**	O	-2.6102510	-1.2439900	-0.4593160
**22**	H	-3.2978110	-1.6696700	-1.0321910
**23**	O	-0.3697990	-2.6626630	-1.3096570
**24**	H	-0.7371330	-3.3312650	-1.9451810
**25**	O	2.9865910	-1.2446850	0.9224370
**26**	H	3.8803730	-1.4404480	1.3072470
**27**	O	2.8714550	1.5680640	0.8232190
**28**	H	3.7479980	1.8695580	1.1784790

Table S2. Geometrical parameters of ATMP.

Number	Element	NA	NB	NC	Bond (Å)	Angle (°)	Dihedral (°)
**1**	P						
**2**	O	1			1.6160600		
**3**	C	1	2		1.9084618	116.0800638	
**4**	H	3	1	2	1.1029368	107.3017224	65.1288115
**5**	H	3	1	2	1.0984951	106.6401429	-178.1552427
**6**	N	3	1	2	1.4537234	111.8831737	-56.4204287
**7**	C	6	3	1	1.4435443	118.3860740	76.9243961
**8**	H	7	6	3	1.1014753	110.9125217	-155.0019522
**9**	H	7	6	3	1.1010006	110.7899748	-33.5553569
**10**	C	6	3	1	1.4313903	121.2804715	-111.5138088
**11**	H	10	6	3	1.1040859	111.6495242	-130.1732778
**12**	H	10	6	3	1.0982050	111.5891783	-7.3947600
**13**	P	10	6	3	1.9461278	115.4084588	112.8901973
**14**	O	13	10	6	1.6086486	121.8687739	-171.6880512
**15**	P	7	6	3	1.9205097	113.1434182	85.9613913
**16**	O	15	7	6	1.6119455	122.0974534	-179.9375180
**17**	O	1	2	3	1.7441830	115.5639566	115.5320421
**18**	H	17	1	2	0.9918327	113.5627587	47.8397982
**19**	O	1	2	17	1.7290123	115.0575400	118.1992113
**20**	H	19	1	2	1.0308257	111.6828776	74.2714456
**21**	O	13	10	6	1.7837019	103.0338932	-44.4263120
**22**	H	21	13	10	0.9910237	113.7775939	175.8096380
**23**	O	13	10	6	1.7517665	98.2500703	62.0343489
**24**	H	23	13	10	0.9929016	115.0868915	173.6188842
**25**	O	15	7	6	1.7577145	98.3366262	54.4787510
**26**	H	25	15	7	0.9925967	114.2654134	176.5930875
**27**	O	15	7	6	1.7573114	99.0229847	-53.8404548
**28**	H	27	15	7	0.9926913	114.1694728	-176.1904397

Table S3. The atomic coordinates for the optimized structure of DTPMP.

Number	Element	X	Y	Z
**1**	C	2.1681480	0.2565120	0.6112290
**2**	H	2.4181720	0.6391220	1.6178710
**3**	H	1.5033650	-0.6053000	0.7529040
**4**	N	3.3508430	-0.2342750	-0.1083810
**5**	C	4.4651830	0.6800440	-0.2723400
**6**	H	5.1727540	0.2804920	-1.0151530
**7**	H	4.1111800	1.6380060	-0.6828300
**8**	C	3.6830090	-1.6341420	0.1054440
**9**	H	4.7702790	-1.7944760	0.1305830
**10**	H	3.2989260	-2.0333490	1.0600280
**11**	P	3.0744930	-2.7396800	-1.2306920
**12**	O	3.4224320	-4.1789440	-1.0545940
**13**	P	5.5168380	1.1602080	1.1806520
**14**	O	6.6009150	2.1312160	0.8402860
**15**	O	1.4732370	-2.3830860	-1.2801320
**16**	H	1.1027380	-2.4684860	-2.1822710
**17**	O	3.5317950	-2.0924850	-2.6478720
**18**	H	4.2534070	-2.6167500	-3.0473930
**19**	O	5.9647180	-0.2902540	1.7458270
**20**	H	6.4486600	-0.2333920	2.5940510
**21**	O	4.5471380	1.6681180	2.3909130
**22**	H	4.5130000	2.6445940	2.4183810
**23**	C	1.4065260	1.3387910	-0.1719080
**24**	H	1.2808910	1.0150510	-1.2227900
**25**	H	1.9931700	2.2688350	-0.1919310
**26**	N	0.1206090	1.6321680	0.4838870
**27**	C	-0.9258070	0.6437970	0.1709140
**28**	H	-1.4207320	0.8601200	-0.7967020
**29**	H	-0.4328500	-0.3299230	0.0557220
**30**	C	-1.9793440	0.5396820	1.2925280
**31**	H	-2.4063750	1.5349520	1.4959080
**32**	H	-1.4849470	0.2137510	2.2205820
**33**	N	-3.1021350	-0.3645450	1.0377700
**34**	C	-0.2997390	3.0222000	0.3990140
**35**	H	-1.1252970	3.2252180	1.0982760
**36**	H	0.5329150	3.6736260	0.7036200
**37**	C	-3.9960360	-0.0055210	-0.0586800
**38**	H	-3.8017310	1.0291330	-0.3744350
**39**	H	-3.8907830	-0.6472220	-0.9510500
**40**	C	-2.9385890	-1.7799740	1.2999240
**41**	H	-3.9341650	-2.2474710	1.3610640
**42**	H	-2.4531990	-1.9241770	2.2771700
**43**	O	-2.0643740	-2.4097840	-1.3553700
**44**	O	-2.6552650	-4.2653120	0.4498250

**45**	H	-2.1258670	-5.0133720	0.1093030
**46**	O	-6.2217390	-1.3774690	1.0364460
**47**	O	-6.4887290	0.5251320	-0.7969220
**48**	H	-7.4620570	0.4574840	-0.7321270
**49**	O	-0.0555580	3.3233430	-2.4453690
**50**	O	-1.0109340	5.2999870	-1.0074790
**51**	H	-0.2127040	5.7745770	-1.3117600
**52**	P	-0.8601860	3.6900760	-1.2408410
**53**	P	-5.7376110	-0.0642240	0.5093690
**54**	P	-1.9977250	-2.8211990	0.0828700
**55**	O	-0.4797180	-2.9458630	0.6415710
**56**	H	0.1916720	-2.8024330	-0.0760790
**57**	O	-5.9087150	1.1546580	1.5760970
**58**	H	-5.8870270	0.8175900	2.4924860
**59**	O	-2.4224050	3.2462420	-1.2611840
**60**	H	-2.8382330	3.4203050	-2.1298180

Table S4. Geometrical parameters of DTPMP.

Number	Element	NA	NB	NC	Bond (Å)	Angle (º)	Dihedral (º)
**1**	C						
**2**	H	1			1.1055454		
**3**	H	1	2		1.0976011	106.9287059	
**4**	N	1	3	2	1.4688356	106.7719510	120.4102576
**5**	C	4	1	3	1.4507293	117.5983458	-172.3625936
**6**	H	5	4	1	1.1009404	109.9497870	-166.8653659
**7**	H	5	4	1	1.1006868	110.0981429	-50.7779042
**8**	C	4	1	5	1.4545388	115.6855301	144.2985696
**9**	H	8	4	1	1.0993157	111.6904239	-143.5661732
**10**	H	8	4	1	1.1036832	113.3148588	-24.2404801
**11**	P	8	4	1	1.8378698	113.3556780	98.2513764
**12**	O	11	8	4	1.4911582	114.6759810	178.8478966
**13**	P	5	4	1	1.8568041	120.6365331	70.8758590
**14**	O	13	5	4	1.4946332	113.6158599	-178.7559080
**15**	O	11	8	4	1.6412265	102.3708493	-54.4585807
**16**	H	15	11	8	0.9789880	112.2123289	147.9804875
**17**	O	11	8	4	1.6236951	107.5487806	49.6160532
**18**	H	17	11	8	0.9773406	110.5439840	107.5746380
**19**	O	13	5	4	1.6198331	101.4243673	54.2838604
**20**	H	19	13	5	0.9782214	112.7853647	-173.0477444
**21**	O	13	5	4	1.6318769	108.9218961	-51.2596821
**22**	H	21	13	5	0.9774586	110.6418006	-99.5081550
**23**	C	1	4	5	1.5377579	112.6101318	68.5780736
**24**	H	23	1	4	1.1067722	109.5061472	46.4173456
**25**	H	23	1	4	1.0997881	109.8925877	-70.7162364
**26**	N	23	1	4	1.4729968	110.2318566	170.6352722

**27**	C	26	23	1	1.4730295	113.0745171	-80.1461778
**28**	H	27	26	23	1.1081639	111.8273151	-82.8909359
**29**	H	27	26	23	1.0974545	107.3697795	33.4530956
**30**	C	27	26	23	1.5423353	112.0880629	152.9566516
**31**	H	30	27	26	1.1019443	109.7525781	55.2545023
**32**	H	30	27	26	1.1008831	109.0426506	-61.1844045
**33**	N	30	27	26	1.4639630	116.0422873	176.7002372
**34**	C	26	23	1	1.4546769	114.6205166	145.3000841
**35**	H	34	26	23	1.1007859	110.8477084	-166.4739520
**36**	H	34	26	23	1.1002060	109.3256379	-49.4789946
**37**	C	33	30	27	1.4595067	116.6493005	-64.8339074
**38**	H	37	33	30	1.0990744	109.8233131	-13.0309508
**39**	H	37	33	30	1.1041660	113.9414738	107.4146571
**40**	C	33	30	27	1.4487620	119.0372603	83.2252893
**41**	H	40	33	30	1.1015730	108.8229498	162.4479365
**42**	H	40	33	30	1.1006397	109.7825118	45.5534800
**43**	O	40	33	30	2.8655713	94.6745142	-91.5431929
**44**	O	40	33	30	2.6419398	150.8047046	-107.8991521
**45**	H	44	40	33	0.9776560	152.8665422	81.9285145
**46**	O	37	33	30	2.8346598	94.1061841	-154.0783627
**47**	O	37	33	30	2.6533210	146.4712572	-123.3659290
**48**	H	47	37	33	0.9778252	154.5376159	-27.9408671
**49**	O	34	26	23	2.8706839	97.6702816	52.3687548
**50**	O	49	34	26	2.6243842	60.3414336	175.8771267
**51**	H	50	49	34	0.9772378	84.1357029	115.9583995
**52**	P	49	34	26	1.4942580	35.0375380	142.6615704
**53**	P	46	37	33	1.4955944	35.3834453	126.1709995
**54**	P	43	40	33	1.4974107	35.3097907	153.1034072
**55**	O	54	43	40	1.6223542	113.1741712	-124.9465597
**56**	H	55	54	43	0.9931557	111.8756926	-4.6921425
**57**	O	53	46	37	1.6287598	113.0971250	-123.3926690
**58**	H	57	53	46	0.9766545	110.7267922	27.1714692
**59**	O	52	49	34	1.6241708	116.1534110	-120.6644609
**60**	H	59	52	49	0.9786398	111.7902510	-43.2926392

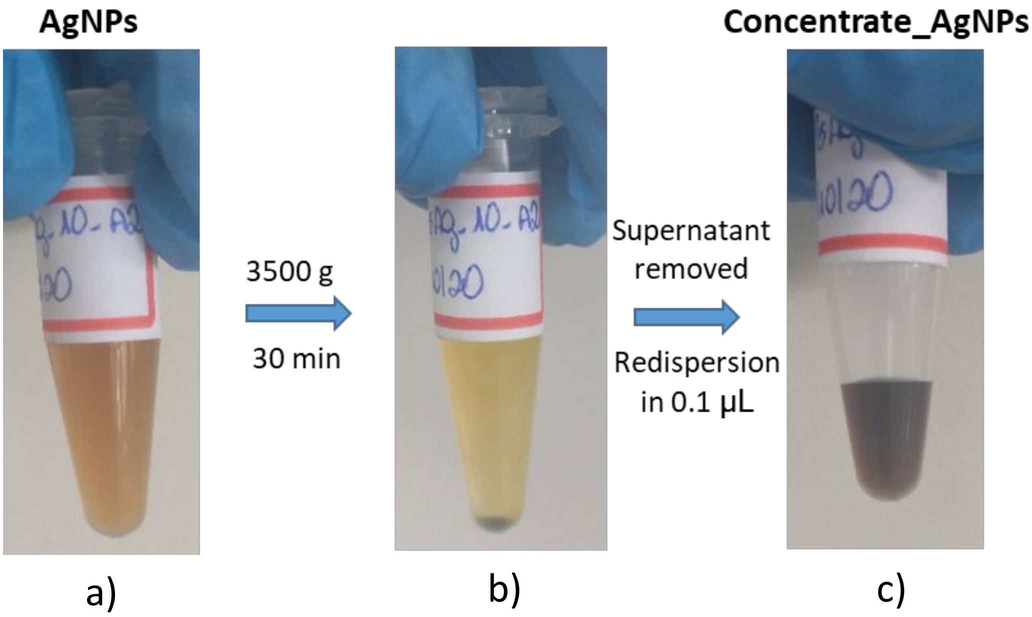

Figure S1. Images of AgNPs colloid: a) before centrifugation, b) after centrifugation, and c) after the supernatant was discarded.

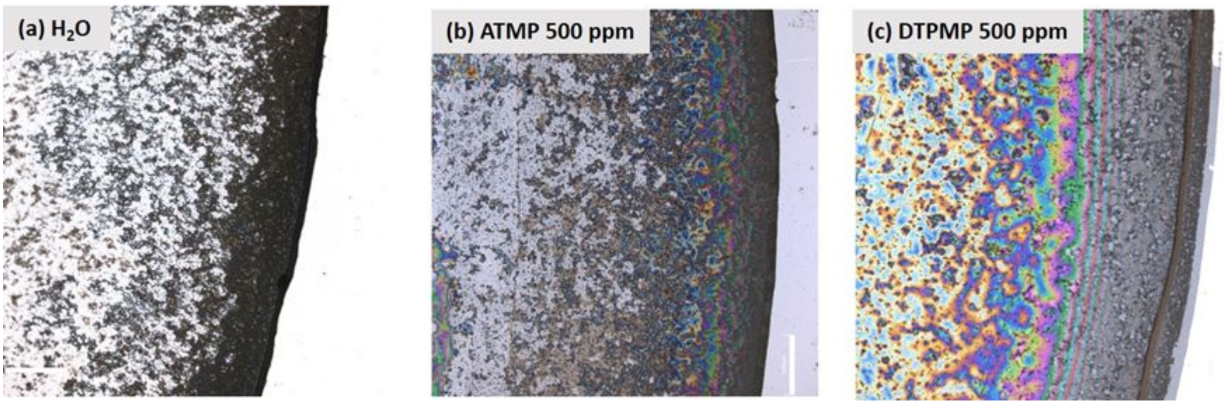

Figure S2. Confocal micrographs of AgNPs droplets after the second droplet of a) H_2O (blank) b) ATMP 500 ppm and c) DTPMP 500 ppm.

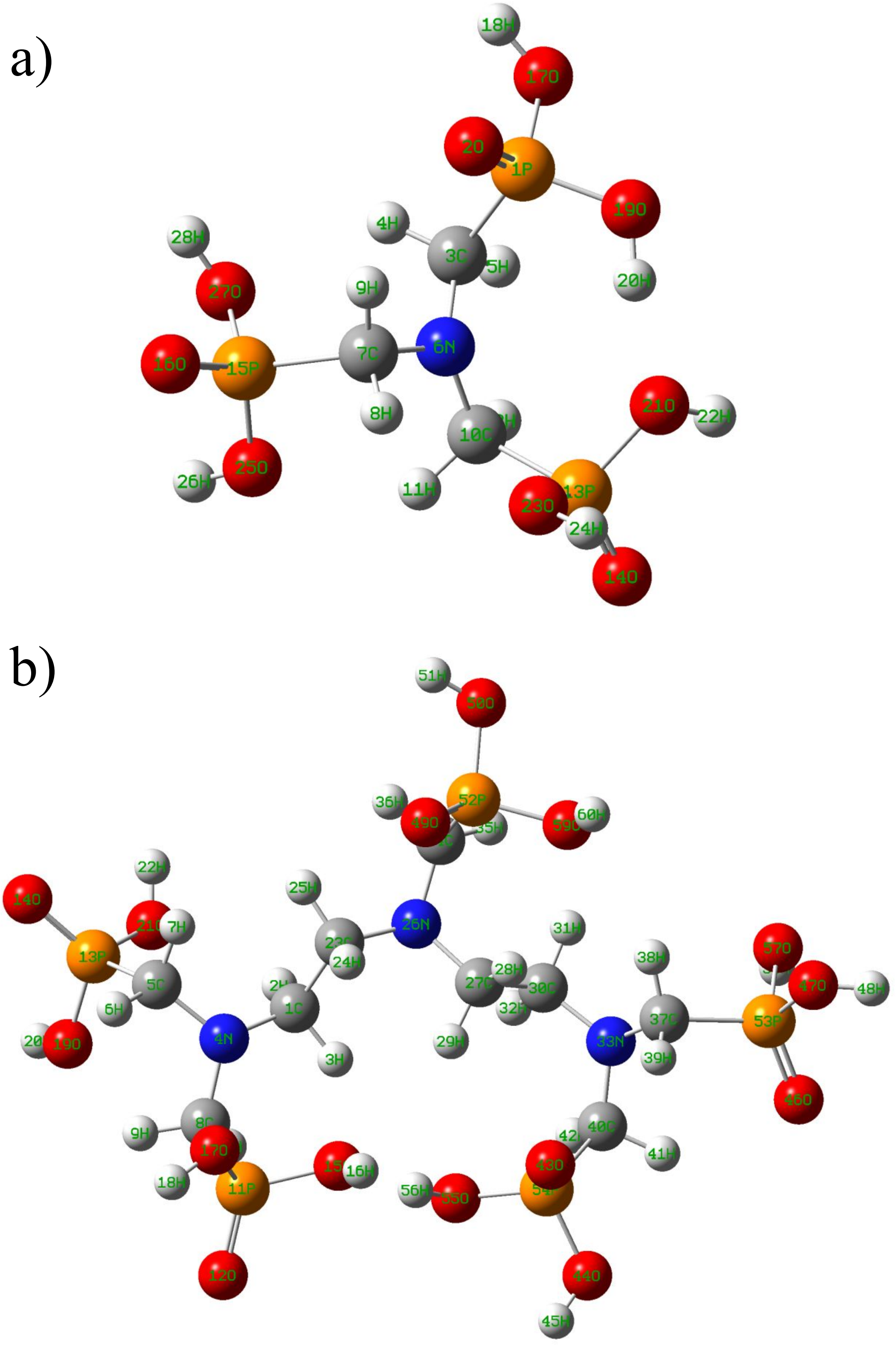

Figure S3. Optimized geometry by DFT of (a) ATMP and (b) DTPMP molecules.

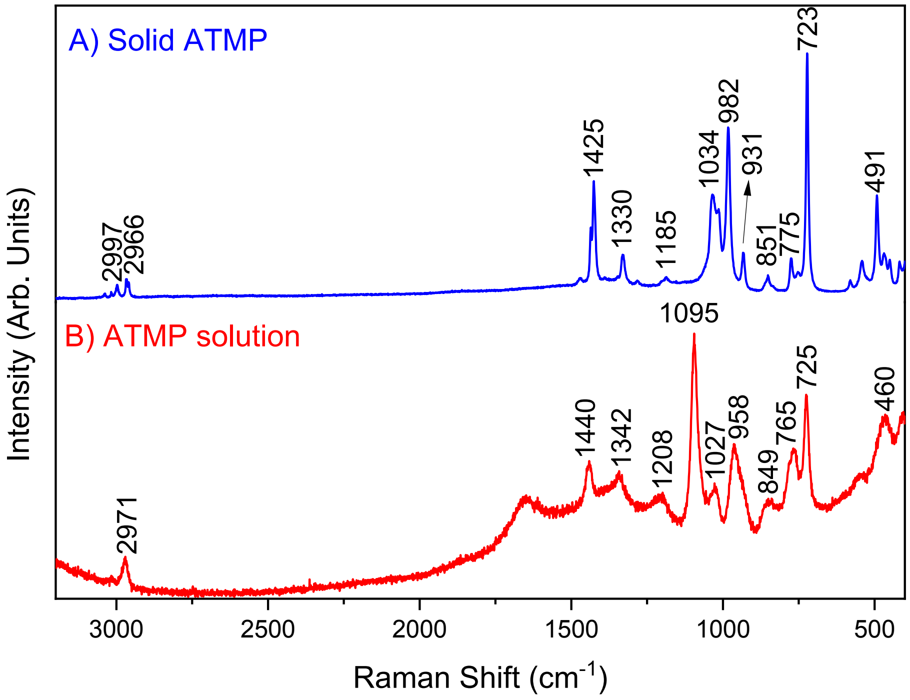

Figure S4. Experimental Raman spectra of A) solid and B) solution (100000 ppm / 3.35 × 10^{-1} mol L^{-1}) phases of ATMP using the source laser at 785 nm.

Table S5. Complete theoretical (BPV86/6–311 +G(2d,p)) and experimental (solid-state and solution (100000 ppm / 3.35 × 10^{-1} mol L^{-1}), Raman (λ_0 = 1064 and 785 nm) and IR bands of ATMP, with the respective assignments based on the potential energy distribution computed with the VEDA 4xx software.

Theoretical ATMP Raman wavenumber (cm^{-1}) DFT/BPV86	Experimental solid ATMP Raman wavenumbers (cm^{-1}) (λ_0 = 1064 nm)	Experimental solid ATMP Raman wavenumbers (cm^{-1}) (λ_0 = 785 nm)	Experimental ATMP solution (3.35 × 10^{-1} mol L^{-1}) Raman wavenumbers (cm^{-1}) (λ_0 = 1064 nm)	Experimental ATMP solution (3.35 × 10^{-1} mol L^{-1}) Raman wavenumbers (cm^{-1}) (λ_0 = 785 nm)	Theoretical ATMP IR wavenumbers (cm^{-1}) DFT/BPV86	Experimental ATMP solution IR wavenumbers (cm^{-1})	Assignments[a]
3693	-	-	-	-	-	-	$\nu(O_{17}H_{18})$ (100%)
3679	-	-	-	-	-	-	$\nu(O_{27}H_{28})$ (100%)
3669	-	-	-	-	-	-	$\nu(O_{23}H_{24})$ (97%)
	-	-	-	-	3664	-	$\nu(O_{21}H_{22})$ (97%)
3661	-	-	-	-	-	-	$\nu(O_{25}H_{26})$ (100%)
3306	-	-	-	-	3306	-	$\nu(O_{19}H_{20})$ (100%)
3043	3029 w	3037 vw	-	-	-	-	$\nu(C_3H_5)$ (88%)
3032	3008 w	3017 vw	-	-	-	-	$\nu(C_7H_8)$ (14%) + $\nu(C_{10}H_{11})$ (19%) + $\nu(C_{10}H_{12})$ (56%)
2980	2994 sh	2997 w	3002	2971 m	-	-	$\nu(C_7H_8)$ (37%) + $\nu(C_7H_9)$ (57%)
2970	2988 s	2966 w	-	-	2970	-	$\nu(C_{10}H_{11})$ (72%) + $\nu(C_{10}H_{12})$ (23%)
2950	2952 vs	2959 w	2956	-	2950	-	$\nu(C_3H_4)$ (90%)
1415	1421 s	1425 s	1426	1440 m	-	1429 vw	$\delta(H_4C_3H_5)$ (34%) + $\delta(H_9C_7H_8)$ (17%) + $\delta(H_{12}C_{10}H_{11})$ (24%)
1393	-	-	-	-	-	-	$\delta(H_4C_3H_5)$ (40%) + $\delta(H_9C_7H_8)$ (35%)
1386	1387 vw	-	-	-	1386	-	$\delta(H_9C_7H_8)$ (27%) + $\delta(H_{12}C_{10}H_{11})$ (45%)
1339	1326 w	1330 w	1327	1342 m	1339	-	$\nu(N_6C_{10})$ (11%) + $\nu(N_6C_3)$ (10%) + $\delta(H_8C_7N_6)$ (39%) + $\tau(H_9C_7N_6C_{10})$ (10%)
1310	1278 vw	-	-	-	1310	-	$\nu(N_6C_7)$ (11%) + $\delta(H_5C_3N_6)$ (22%) + $\delta(H_{11}C_{10}N_6)$ (13%) + $\delta(H_{12}C_{10}H_{11})$ (10%) + $\tau(H_{12}C_{10}N_6C_3)$ (14%)
1283	-	-	-	-	-	-	$\delta(H_{11}C_{10}N_6)$ (30%) + $\tau(H_8C_7N_6C_{10})$ (11%) + $\tau(H_9C_7N_6C_{10})$ (17%)
1273	-	-	-	-	-	-	$\tau(H_8C_7N_6C_{10})$ (20%) + $\tau(H_9C_7N_6C_{10})$ (15%) + $\tau(H_{11}C_{10}N_6C_3)$ (14%) + $\tau(H_{12}C_{10}N_6C_3)$ (20%)
1264	-	-	-	-	1264	-	$\delta(H_4C_3H_5)$ (11%) + $\tau(H_4C_3N_6C_{10})$ (43%) + $\tau(H_5C_3N_6C_{10})$ (24%)
1201	1196 sh	1200 sh	1213	1208 w	-	-	$\nu(P_1O_2)$ (22%) + $\nu(P_{13}O_{14})$ (35%) + $\delta(H_{11}C_{10}N_6)$ (12%)
1192	-	-	-	-	1192	1182 s	$\nu(P_{13}O_{14})$ (42%) + $\nu(P_1O_2)$ (26%)
1185	1184 w	1185 vw	1181	-	-	-	$\nu(P_{15}O_{16})$ (81%)
1170	-	-	-	-	-	-	$\delta(H_5C_3N_6)$ (14%) + $\delta(H_8C_7N_6)$ (16%) + $\nu(P_1O_2)$ (31%)
1129	-	-	-	-	1129	-	$\delta(H_{20}O_{19}P_1)$ (82%)
1108	-	-	-	-	1108	-	$\delta(H_5C_3N_6)$ (10%) + $\nu(N_6C_7)$ (36%)
1094	-	-	-	-	1094	-	$\nu(N_6C_{10})$ (25%) + $\nu(N_6C_3)$ (27%) + $\delta(H_8C_7N_6)$ (10%)
1034	1034 m	1034 m	1082	1095 vs	-	1078 vs	$\delta(H_{22}O_{21}P_{13})$ (80%)
1014	1011 sh	1014 m	1012	1027 m	1014	-	$\delta(H_{28}O_{27}P_{15})$ (80%)
1006	978 vs	982 s	949	958 s	-	-	$\delta(H_{24}O_{23}P_{13})$ (59%) + $\delta(H_{26}O_{25}P_{15})$ (23%)
1000	929 w	-	923 sh	-	1000	929 m	$\delta(H_{24}O_{23}P_{13})$ (26%) + $\delta(H_{26}O_{25}P_{15})$ (57%)
877	-	-	-	-	877	-	$\nu(P_1O_{19})$ (20%)
857	-	-	-	-	857	-	$\nu(P_1O_{19})$ (45%)
831	849 vw	862 w	-	-	-	-	$\nu(N_6C_7)$ (11%) + $\nu(N_1C_{19})$ (11%) + $\nu(P_1O_{17})$ (20%)
823	-	-		-	823	-	$\nu(N_6C_{10})$ (13%) + $\nu(P_{15}O_{25})$ (17%) + $\tau(H_5C_3N_6C_{10})$ (11%)
808		-		-	808	-	$\nu(P_{13}O_{23})$ (23%) + $\nu(P_1O_{17})$ (49%)
800	832 vw	851 sh		849 w	-	-	$\nu(P_{15}O_{27})$ (54%) + $\nu(P_{15}C_7)$ (12%)
781		-	-	-	781	-	$\nu(P_{13}O_{23})$ (15%) + $\nu(P_{15}C_{25})$ (24%)
770		-	-	-	-	-	$\nu(P_{13}O_{21})$ (53%) + $\nu(P_{13}C_{10})$ (11%)

694	774 w	775 w	756	766 m	-	752 vw	$\nu(P_1C_3)$ (44%) + $\delta(P_1C_3N_6)$ (10%)
664		754 vw		-	-		$\nu(O_{21}H_{22})$ (97%)
650	720 vs	721 vs	713	725 s	-	713 vw	$\nu(P_{15}O_{27})$ (15%) + $\nu(P_{15}C_7)$ (48%) + $\delta(P_{15}C_7N_6)$ (14%)
608	-	-	-	-	608	-	$\tau(H_{20}O_{19}P_1C_3)$ (11%)
488	581 vw	-	598	-	488	-	$\delta(P_1C_3N_6)$ (11%) + $\gamma(O_2C_3O_{19}P_1)$ (26%)
451	541 vw	-	-	-	451	-	$\delta(O_{14}P_{13}O_{23})$ (11%) + $\delta(O_2P_1O_{19})$ (11%) + $\delta(O_{19}P_1O_{17})$ (10%) + $\delta(O_{21}P_{13}O_{14})$ (13%)
406	-	-	-	-	406	-	$\delta(C_3N_6C_{10})$ (16%) + $\delta(O_{16}P_{15}O_{27})$ (20%) + $\delta(O_{25}P_{15}O_{16})$ (21%)
400	491 w	491 m	484 sh	460 m	-	-	$\delta(O_{21}P_{13}O_{14})$ (19%) + $\delta(C_7N_6C_3)$ (21%)
387	-	-	-	-	-	-	$\tau(H_{22}O_{21}P_{13}C_{10})$ (11%) + $\delta(O_{14}P_{13}O_{23})$ (18%) + $\delta(O_{23}P_{13}O_{21})$ (30%)
368	-	-	-	-	368	-	$\delta(O_{19}P_1O_{17})$ (25%) + $\delta(O_{17}P_1O_2)$ (19%) + $\tau(H_{18}O_{17}P_1C_3)$ (15%)
318	-	-	-	-	-	-	$\gamma(O_{19}C_3O_{17}P_1)$ (18%)
313	-	-	-	-	313	-	$\delta(O_{17}P_1O_2)$ (10%) + $\tau(H_{22}O_{21}P_{13}C_{10})$ (20%) + $\gamma(O_{14}C_{10}O_{23}P_{13})$ (14%)
282	-	-	-	-	-	-	$\tau(H_{22}O_{21}P_{13}C_{10})$ (36%)
270	-	-	-	-	270	-	$\tau(H_{28}O_{27}P_{15}C_7)$ (35%) + $\gamma(O_{16}C_7O_{27}P_{15})$ (15%) + $\gamma(O_{19}C_3O_{17}P_1)$ (16%)
261	-	-	-	-	-	-	$\tau(H_{18}O_{17}P_1C_3)$ (10%) + $\tau(H_{28}O_{27}P_{15}C_7)$ (18%) + $\gamma(O_{19}C_3O_{17}P_1)$ (20%)
237	-	-	-	-	-	-	$\delta(O_{17}P_1O_2)$ (10%) + $\tau(H_{18}O_{17}P_1C_3)$ (25%) + $\gamma(O_{23}C_{10}O_{21}P_{13})$ (23%)
224	-	-	-	-	-	-	$\delta(O_{17}P_1O_2)$ (10%) + $\tau(H_{24}O_{23}P_{13}C_{10})$ (34%)
206	-	-	-	-	-	-	$\tau(H_{24}O_{23}P_{13}C_{10})$ (34%) + $\tau(H_{26}O_{25}P_{15}C_7)$ (27%)
183	-	-	-	-	183	-	$\delta(O_{14}P_{13}O_{23})$ (10%) + $\delta(O_{23}P_{13}O_{21})$ (12%) + $\tau(H_{24}O_{23}P_{13}C_{10})$ (30%) + $\gamma(O_{14}C_{10}O_{23}P_{13})$ (11%)
172	-	-	-	-	-	-	$\tau(H_{26}O_{25}P_{15}C_7)$ (31%) + $\gamma(O_{27}C_7O_{25}P_{15})$ (10%)
138	-	-	-	-	-	-	$\delta(P_1C_3N_6)$ (15%) + $\delta(P_{15}C_7N_6)$ (22%) + $\gamma(O_{27}C_7O_{25}P_{15})$ (15%)
127	-	-	-	-	-	-	$\gamma(O_{14}C_{10}O_{23}P_{13})$ (13%) + $\delta(O_{23}P_{13}O_{21})$ (11%) + $\delta(P_{13}C_{10}N_6)$ (23%)
120	-	-	-	-	-	-	$\tau(O_{25}P_{15}C_7N_6)$ (14%) + $\tau(O_{17}P_1C_3N_6)$ (18%) + $\tau(O_{21}P_{13}C_{10}N_6)$ (10%) + $\tau(P_{15}C_7N_6C_3)$ (19%)
94	-	-	-	-	-	-	$\delta(P_{15}C_7N_6)$ (11%) + $\tau(P_1C_3N_6C_{10})$ (13%) + $\tau(P_{13}C_{10}N_6C_3)$ (15%) + $\gamma(C_7C_{10}C_3N_6)$ (20%)
74	-	-		-	-	-	$\gamma(C_7C_{10}C_3N_6)$ (31%) + $\delta(P_1C_6N_6)$ (10%)
60	-	-	-	-	-	-	$\tau(P_{15}C_7N_6C_{10})$ (27%) + $\tau(O_{21}P_{13}C_{10}N_6)$ (27%) + $\tau(O_{17}P_1C_3N_6)$ (23%)

ν, stretching; δ, in-plane deformation; τ, torsional; γ, out-of-plane;

vs, very strong; s, strong; m, medium; vw, very weak; w, weak; sh, shoulder.

[a]All assignments include internal coordinates that contribute 10% or more to the PED.

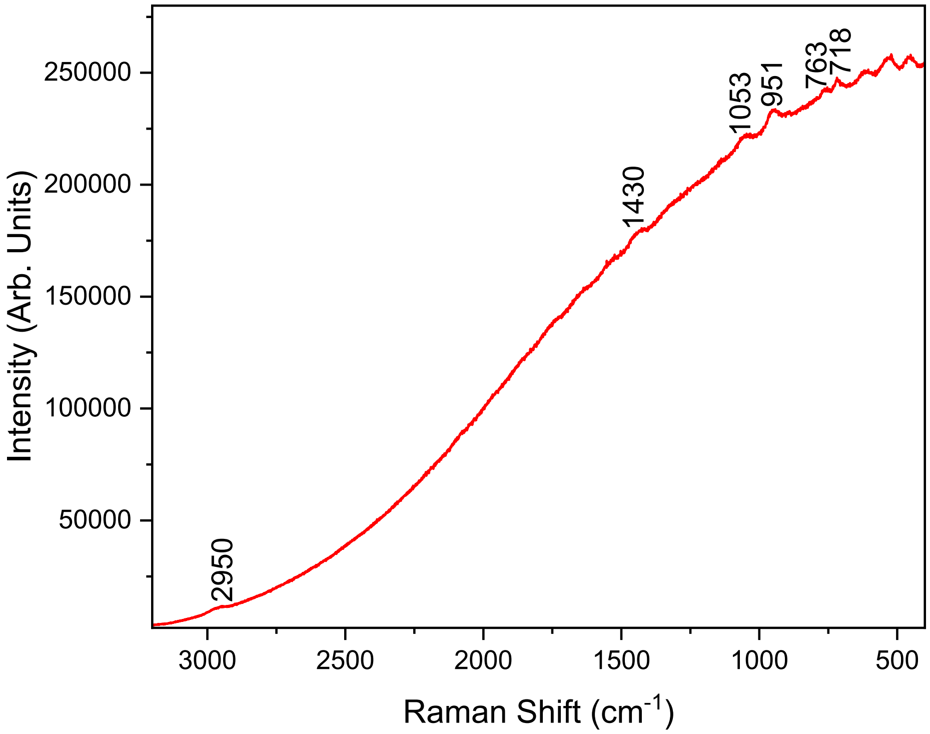

Figure S5. Raman spectrum of DTPMP solution (500000 ppm / 8.72 × 10^{-1} mol L^{-1}) using the laser source at 785 nm.

Table S6. Complete theoretical (BPV86/6–311 +G(2d,p)) and experimental (solid-state and solution (500000 ppm / 8.72 × 10^{-1} mol $L^{-1)}$, Raman (λ_0 = 1064 and 785 nm) and IR bands of DTPMP, with the respective assignments based on the potential energy distribution computed with the VEDA 4xx software.

Theoretical DTPMP Raman wavenumbers (cm^{-1}) DFT/BPV86	Experimental DTPMP (8.72 × 10^{-1} mol L^{-1}) SERS wavenumbers (cm^{-1}) (1064 nm)	Experimental DTPMP solution (8.72 × 10^{-1} mol L^{-1}) Raman wavenumbers (cm^{-1}) (785 nm)	Theoretical DTPMP IR wavenumbers (cm^{-1}) DFT/BPV86	Experimental DTPMP (8.72 × 10^{-1} mol L^{-1}) IR wavenumbers (cm^{-1})	Assigments[a]
3692	-	-	-	-	ν($O_{57}H_{58}$) (100%)
3682	-	-	3682	-	ν($O_{50}H_{51}$) (100%)
3671	-	-	3671	-	ν($O_{47}H_{48}$) (100%)
3664	-	-	3664	-	ν($O_{19}H_{20}$) (100%)
3657	-	-	-	-	ν($O_{59}H_{60}$) (100%)
3347	-	-	3347	-	ν($O_{55}H_{56}$) (100%)
-	-	-	3037	-	ν(C_1H_3) (24%) + ν($C_{27}H_{29}$) (63 %)
3028	-	-	-	-	ν($C_{34}H_{35}$) (34%) + ν($C_{34}H_{36}$) (57%)
3004	-	-	3004	-	ν($C_{23}H_{25}$) (84%)
2978	2937 vs	-	2978	-	ν($C_{34}H_{35}$) (61%) + ν($C_{34}H_{36}$) (37%)
2969	-	-	-	-	ν($C_{40}H_{41}$) (58%) + ν($C_{40}H_{42}$) (40%)
2958	2954 sh	2950 vw	2958	-	ν($C_{30}H_{31}$) (72%) + ν($C_{30}H_{32}$) (24%)
2954	-	-	-	-	ν(C_8H_9) (12%) + ν(C_8H_{10}) (87%)

2942	-	-	-	-	ν($C_{37}H_{39}$) (88%)
2931	-	-	2931	-	ν(C_1H_2) (89%)
2910	-	-	2910	-	ν($C_{23}H_{24}$) (89%)
2897	-	-	2897	-	ν($C_{27}H_{28}$) (97%)
-	-	-	1454	-	δ($H_2C_1H_3$) (37%) + δ($H_{25}C_{23}H_{24}$) (35%)
1445	-	-	1445	-	δ($H_{25}C_{23}H_{24}$) (20%) + δ($H_{29}C_{27}H_{28}$) (49%)
1437	1456 s	-	-	-	δ($H_2C_1H_3$) (38%) + δ($H_{25}C_{23}H_{24}$) (21%) + δ($H_{32}C_{30}H_{31}$) (11%)
1429	1431 s	1430 vw	-	-	δ($H_{39}C_{37}H_{38}$) (10%) + δ($H_{29}C_{27}H_{28}$) (13%) + δ($H_{32}C_{30}H_{31}$) (47%)
1403	-	-	1403	-	δ($H_{39}C_{37}H_{38}$) (60%) + δ($H_{32}C_{30}H_{31}$) (14%)
1394	-	-	-	-	δ($H_{36}C_{34}H_{35}$) (56%)
1385	-	-	1385	-	δ($H_{42}C_{40}H_{41}$) (58%) + δ($H_7C_5H_6$) (37%)
1369	-	-	1369	-	τ($H_2C_1N_4C_8$) (10%) + δ($H_9C_8H_{10}$) (20%)
1335	-	-	1335	-	δ($H_{38}C_{37}N_{33}$) (10%) + δ($H_{41}C_{40}N_{33}$) (43%)
1326	-	-	1326	-	τ($H_7C_5N_4C_8$) (11%) + δ($H_3C_1C_{23}$) (10%) + δ($H_6C_5N_4$) (32%)
1304	-	-	-	-	δ($H_{35}C_{34}N_{26}$) (10%) + δ($H_{24}C_{23}N_{26}$) (10%)
1294	-	-	1294	-	δ($H_{28}C_{27}C_{30}$) (19%)
1283	-	-	-	-	τ($H_{41}C_{40}N_{33}C_{30}$) (28%) + τ($H_{42}C_{40}N_{33}C_{30}$) (27%) + τ($H_7C_5N_4C_8$) (22%)
1257	-	-	1257	-	τ($H_{38}C_{37}N_{33}C_{30}$) (24%) + τ($H_{39}C_{37}N_{33}C_{30}$) (30%) +
-	-	-	1244	-	τ($H_3C_1N_4C_8$) (11%) + δ($H_{10}C_8N_4$) (24%) + δ($H_{24}C_{23}N_{26}$) (27%)
1188	1224 sh	-	1188	1171 s	ν($P_{11}O_{12}$) (23%) + ν($P_{53}O_{46}$) (20%) + ν($P_{54}O_{43}$) (27%) + δ($H_{56}O_{55}P_{54}$) (10%)
1179	1198 w	-	-	-	ν($P_{13}O_{14}$) (74%)
1159	-	-	-	-	δ($H_{38}C_{37}N_{33}$) (12%)
1137	-	-	-	-	ν($N_{33}C_{40}$) (10%) + ν($N_{33}C_{37}$) (10%)
-	-	-	1132	-	ν($P_{54}O_{43}$) (26%) + δ($H_{56}O_{55}P_{54}$) (53%)
1105	-	-	-	-	ν(N_4C_5) (17%) + ν($N_{26}C_{34}$) (11%)
-	-	-	1095	-	δ($H_{38}C_{37}N_{33}$) (11%) + ν($N_{33}C_{30}$) (35%)
1092	-	-	-	1075 s	ν(N_4C_1) (33%) + ν(N_4C_8) (11%) + δ($H_6C_5N_4$) (10%)
1073	-	-	-	-	ν($N_{26}C_{23}$) (19%) + ν($N_{26}C_{27}$) (22%) + δ($H_{35}C_{34}N_{26}$) (10%)
1039	-	-	-	-	ν(C_1C_{23}) (12%)
1032	1066 m	1053 m	-	-	ν(C_1C_{23}) (39%) + δ($H_{16}O_{15}P_{11}$) (21%)
1014	-	-	-	-	δ($H_{18}O_{17}P_{11}$) (80%)
-	-	-	1010	1010 s	δ($H_{20}O_{19}P_{13}$) (10%) + δ($H_{22}O_{21}P_{13}$) (77%)
1007	-	-	-	-	δ($H_{60}O_{59}P_{52}$) (63%) + δ($H_{51}O_{50}P_{52}$) (20%)
996	953 s	951 s	996	940 m	δ($H_{51}O_{50}P_{52}$) (55%) + δ($H_{60}O_{59}P_{52}$) (19%)
851	-	-	-	-	ν(N_4C_5) (14%)
-	-	-	845	-	ν($P_{54}O_{55}$) (12%)
821	-	-		-	ν($P_{13}O_{19}$) (10%) + ν($P_{11}O_{17}$) (10%) + ν($N_{33}C_{30}$) (11%)
-	-	-	814	-	ν($P_{53}O_{57}$) (23%) + ν($P_{53}O_{47}$) (45%)
809	-	-	-	-	ν($P_{11}O_{17}$) (17%)
800	-	-	-	-	ν($P_{13}O_{21}$) (57%) + ν($P_{13}C_5$) (11%)
794	-	-	794	-	ν($P_{52}O_{59}$) (25%) + ν($P_{52}O_{50}$) (48%)
779	-	-	779	-	ν($P_{52}O_{59}$) (37%) + ν($P_{52}O_{50}$) (30%)
765	-	-		-	ν($P_{11}O_{15}$) (55%)
691	766 m	763 m	690	754 vw	ν($P_{53}O_{57}$) (15%) + ν($P_{53}C_{57}$) (45%) + δ($P_{53}C_{37}N_{33}$) (12%)
675	-	-	675	-	ν($P_{11}C_8$) (39%) + δ($P_{11}C_8N_4$) (11%) + ν($P_{11}O_{17}$) (12%) + ν($P_{11}O_{15}$) (17%)

-	-	-	653	-	$\nu(P_{52}C_{34})$ (44%) + $\delta(P_{52}C_{34}N_{26})$ (20%)
649	717 m	718 s	-	-	$\nu(P_{54}C_{40})$ (52%) + $\delta(P_{54}C_{40}N_{33})$ (16%)
636	-	-	-	-	$\nu(P_{13}C_{5})$ (47%) + $\delta(P_{13}C_{5}N_{4})$ (15%) + $\nu(P_{13}O_{21})$ (13%)
-	-	-	608	-	$\tau(H_{56}O_{55}P_{54}C_{40})$ (89%)
541	-	-	-	-	$\gamma(O_{49}C_{34}O_{59}P_{52})$ (12%) + $\nu(P_{52}C_{34})$ (14%) + $\delta(C_{1}C_{23}N_{26})$ (10%)
504	-	-	504	-	$\delta(P_{11}C_{8}N_{4})$ (10%) + $\delta(P_{13}C_{5}N_{4})$ (11%)
492	-	-	492	-	$\delta(P_{54}C_{40}N_{33})$ (17%) + $\gamma(O_{43}C_{40}O_{55}P_{54})$ (17%)
-	-	-	442	-	$\delta(C_{34}N_{26}C_{27})$ (11%)
428	-	-	-	-	$\delta(O_{21}P_{13}O_{14})$ (12%) + $\delta(O_{15}P_{11}O_{12})$ (15%)
411	440 s	-	411	-	$\delta(O_{43}P_{54}O_{55})$ (16%)
408	-	-			$\delta(O_{17}P_{11}O_{15})$ (11%)
383	-	-	383	-	$\delta(O_{57}P_{53}O_{17})$ (10%)
374	-	-		-	$\delta(O_{50}P_{52}O_{49})$ (13%)
366	-	-	366	-	$\delta(O_{43}P_{53}O_{57})$ (40%) + $\delta(O_{55}P_{54}O_{44})$ (23%) + $\gamma(O_{55}C_{40}O_{44}P_{54})$ (21%)
357	-	-	-	-	$\delta(O_{12}P_{11}O_{17})$ (40%) + $\delta(O_{15}P_{11}O_{12})$ (16%) + $\gamma(O_{12}C_{8}O_{17}P_{11})$ (14%)
346	-	-	-	-	$\delta(O_{14}P_{13}O_{19})$ (16%)
335	-	-	-	-	$\delta(C_{40}N_{33}C_{37})$ (14%)
267	-	-	-	-	$\gamma(O_{59}C_{34}O_{50}P_{52})$ (17%)
245	-	-	245	-	$\delta(O_{50}P_{52}O_{49})$ (11%) + $\tau(H_{60}O_{59}P_{52}C_{34})$ (29%)
236	-	-	-	-	$\tau(H_{16}O_{15}P_{11}C_{8})$ (19%)
-	-	-	233	-	$\tau(H_{48}O_{47}P_{53}C_{37})$ (15%)
226	-	-	-	-	$\tau(H_{22}O_{21}P_{13}C_{5})$ (18%) + $\gamma(O_{12}C_{8}O_{17}P_{11})$ (11%)
-	-	-	217	-	$\tau(H_{45}O_{44}P_{54}C_{40})$ (17%) + $\gamma(O_{55}C_{40}O_{44}P_{54})$ (12%)
214	-	-	-	-	$\tau(H_{18}O_{17}P_{11}C_{8})$ (27%) + $\gamma(O_{17}C_{8}O_{15}P_{11})$ (15%)
184	-	-	184	-	$\tau(H_{48}O_{47}P_{53}C_{37})$ (16%)
164	-	-	164	-	$\tau(H_{20}O_{19}P_{13}C_{5})$ (43%)
148	-	-	-	-	$\delta(O_{55}P_{54}O_{44})$ (11%) + $\tau(H_{45}O_{44}P_{54}C_{40})$ (22%)
138	-	-	138	-	$\tau(H_{45}O_{44}P_{54}C_{40})$ (12%)
132	-	-	-	-	$\delta(N_{33}C_{30}C_{27})$ (11%)
111	-	-	-	-	$\delta(P_{11}C_{8}N_{4})$ (13%) + $\delta(P_{13}C_{5}N_{4})$ (23%)
104	-	-	104	-	$\delta(P_{54}C_{40}N_{33})$ (14%) + $\delta(P_{53}C_{37}N_{33})$ (12%)
88	-	-	-	-	$\tau(O_{44}P_{40}C_{33}N_{30})$ (16%)
-	-	-	41	-	$\tau(P_{54}C_{40}N_{33}C_{30})$ (15%) + $\tau(O_{44}P_{54}C_{40}N_{33})$ (10%)

ν, stretching; δ, in-plane deformation; τ, torsional; γ, out-of-plane.

vs, very strong; s, strong; m, medium; w, weak; sh, shoulder.

[a]All assignments include internal coordinates that contribute 10% or more to the PED.

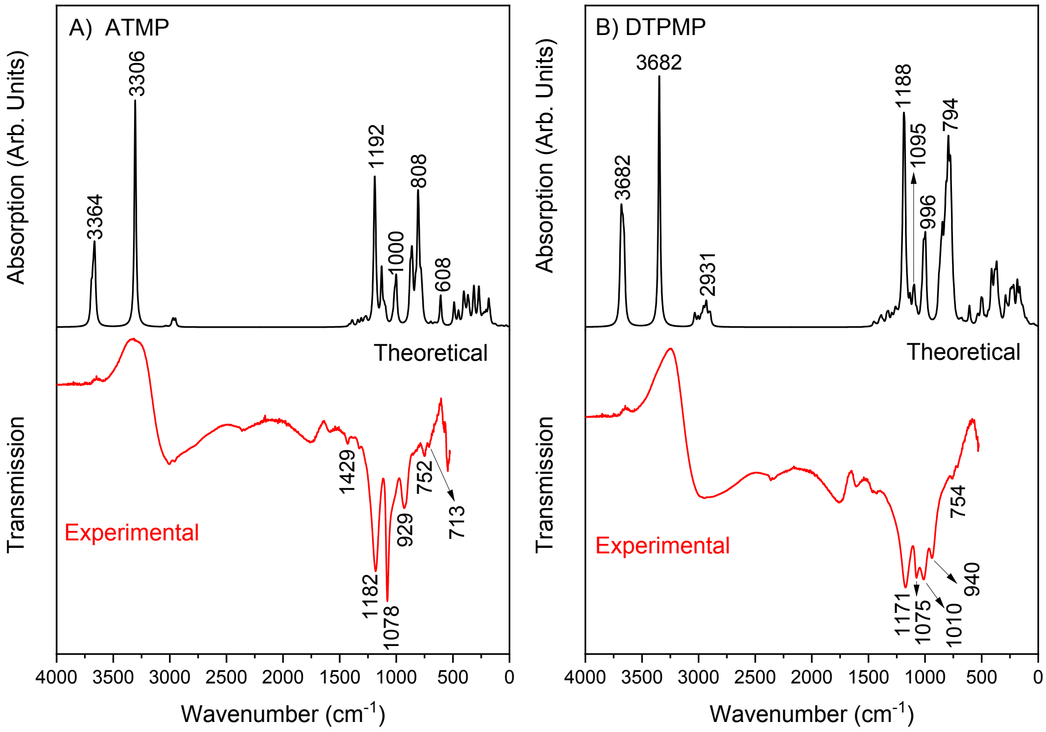

Figure S6. Theoretical and experimental IR spectra of A) ATMP and B) DTPMP molecules.

Table S7. The atomic coordinates for the optimized structure of ATMP model adsorption of N-Ag_{10}.

Number	Element	X	Y	Z
**1**	P	-3,4899910	-1,5611420	-1,5451940
**2**	O	-3,0077990	-2,1671630	-0,2499110
**3**	C	-3,8418320	0,2482090	-1,4052800
**4**	H	-2,8636020	0,7589710	-1,3388790
**5**	H	-4,3169510	0,5747640	-2,3408030
**6**	N	-4,6974210	0,5358310	-0,2660250
**7**	C	-4,0616260	0,6419280	1,0309800
**8**	H	-4,7981570	0,4849550	1,8338150
**9**	H	-3,3056350	-0,1513840	1,1353670
**10**	C	-5,9691020	1,1988250	-0,4774050
**11**	H	-6,1062190	2,0515640	0,2060410
**12**	H	-6,0470380	1,6006480	-1,4982530
**13**	P	-7,4231170	0,0914990	-0,2503610
**14**	O	-8,7704850	0,6823280	-0,4907080
**15**	P	-3,1930550	2,2227560	1,4445400
**16**	O	-2,2735670	2,0995380	2,6325470

**17**	O	-2,4750920	-1,7680140	-2,7892650
**18**	H	-1,5477100	-1,6312150	-2,4816190
**19**	O	-4,7868360	-2,2568430	-2,1812240
**20**	H	-5,6348840	-1,9238480	-1,7756450
**21**	O	-7,1514000	-1,2159220	-1,2326800
**22**	H	-7,8096580	-1,2163120	-1,9573180
**23**	O	-7,1120160	-0,5199890	1,2073160
**24**	H	-7,7384720	-1,2216340	1,4751530
**25**	O	-4,4422290	3,2384420	1,6042750
**26**	H	-4,1662890	4,1714780	1,7055260
**27**	O	-2,4819460	2,8187760	0,1151650
**28**	H	-1,5689350	2,4459290	0,0056240
**29**	Ag	4,1096520	-0,7391190	0,1746850
**30**	Ag	1,7563030	-1,5591350	-1,1927520
**31**	Ag	2,4707500	1,4439830	0,9571670
**32**	Ag	-0,6826060	-1,3549820	0,1850620
**33**	Ag	-0,1414720	0,9342870	1,8918200
**34**	Ag	1,7410970	-1,1769970	1,6788460
**35**	Ag	2,9804260	0,9023860	-1,8400080
**36**	Ag	0,3372220	0,9635810	-0,9310650
**37**	Ag	0,9307960	-3,6469810	0,6755680
**38**	Ag	1,8940930	3,3645840	-0,9962480

Table S8. Geometrical parameters of ATMP model adsorption of N-Ag_{10}.

**Number**	**Element**	**NA**	**NB**	**NC**	**Bond (Å)**	**Angle (º)**	**Dihedral (º)**
**1**	P						
**2**	O	1			1.5091483		
**3**	C	1	2		1.8485451	112.8862026	
**4**	H	3	1	2	1.1055410	106.7583925	72.7986012
**5**	H	3	1	2	1.0988992	107.9822051	-172.1649732
**6**	N	3	1	2	1.4534995	111.4106624	-50.3621482
**7**	C	6	3	1	1.4483487	117.2577073	82.2737983
**8**	H	7	6	3	1.1007554	110.4239013	-158.9521836
**9**	H	7	6	3	1.1008011	109.4876051	-41.7389497
**10**	C	6	3	1	1.4496259	119.5112716	-121.5365985
**11**	H	10	6	3	1.1013915	111.8902812	-130.9466327
**12**	H	10	6	3	1.0998483	111.3828148	-11.8467255
**13**	P	10	6	3	1.8417056	113.5544507	107.9251502
**14**	O	13	10	6	1.4907200	117.0906222	-178.1402647
**15**	P	7	6	3	1.8505309	117.9530250	79.2280777
**16**	O	15	7	6	1.5073160	113.1180170	-164.6868995
**17**	O	1	2	3	1.6188047	114.0774199	123.2547522
**18**	H	17	1	2	0.9866090	109.3871483	-41.2290794
**19**	O	1	2	17	1.6032283	115.1314697	112.7812993
**20**	H	19	1	2	0.9972790	112.4341518	82.7682553

**21**	O	13	10	6	1.6577486	105.7675555	-53.3438446
**22**	H	21	13	10	0.9789811	109.1908976	-112.6840414
**23**	O	13	10	6	1.6110629	100.7950480	52.3709294
**24**	H	23	13	10	0.9780028	113.3532728	-173.8121914
**25**	O	15	7	6	1.6178903	101.3006720	69.9908871
**26**	H	25	15	7	0.9782386	113.0292800	-171.9874315
**27**	O	15	7	6	1.6211581	109.6754674	-36.0282517
**28**	H	27	15	7	0.9922717	110.8537942	-87.8087359
**29**	Ag	2	1	19	7.2717055	106.4984621	153.8951687
**30**	Ag	29	2	1	2.8426329	26.4846270	-44.0424467
**31**	Ag	29	2	1	2.8397557	66.5578208	80.6028065
**32**	Ag	2	1	19	2.5010722	108.4158398	164.5059663
**33**	Ag	16	15	7	2.5401408	108.6441684	77.5008731
**34**	Ag	31	29	2	2.8147380	60.2913218	39.0575132
**35**	Ag	30	29	2	2.8242704	59.9999049	110.3784024
**36**	Ag	32	2	1	2.7679456	122.9848061	22.2260049
**37**	Ag	34	31	29	2.7863911	143.9488723	-65.4757452
**38**	Ag	31	29	2	2.7994770	117.0023659	-95.1229028

Table S9. The atomic coordinates for the optimized structure of DTPMP model adsorption of N-Ag_{10}.

**Number**	**Element**	**X**	**Y**	**Z**
**1**	C	4.8926120	2.3075720	-1.1174600
**2**	H	5.6848960	2.6702360	-1.8005040
**3**	H	4.4450060	1.4180500	-1.5829690
**4**	N	3.8018880	3.2782270	-0.9419570
**5**	C	4.1063880	4.5212550	-0.2563060
**6**	H	3.1720020	5.0707410	-0.0655710
**7**	H	4.5395850	4.3123150	0.7342940
**8**	C	2.8955510	3.3914480	-2.0817930
**9**	H	2.9476530	4.3666940	-2.5946300
**10**	H	3.1389030	2.6376340	-2.8446440
**11**	P	1.1272800	3.1312200	-1.6662430
**12**	O	0.1972610	3.3553900	-2.8145800
**13**	P	5.2604620	5.7735240	-0.9958310
**14**	O	5.4292230	6.9968370	-0.1533230
**15**	O	1.1333650	1.6560410	-0.9892400
**16**	H	0.2192360	1.3747710	-0.6651750
**17**	O	0.7995150	4.0663900	-0.3705570
**18**	H	0.2799280	4.8475050	-0.6449670
**19**	O	4.6415460	5.9727770	-2.4807140
**20**	H	5.2112010	6.5223000	-3.0562680
**21**	O	6.6742240	5.0387030	-1.3512380
**22**	H	7.3443490	5.2405640	-0.6689200
**23**	C	5.5315920	1.8815030	0.2135640

**24**	H	4.7453900	1.7791520	0.9868350
**25**	H	6.2311300	2.6526670	0.5658600
**26**	N	6.2877560	0.6323560	0.0244930
**27**	C	5.4322880	-0.5671520	0.0422100
**28**	H	5.3029040	-0.9627560	1.0686810
**29**	H	4.4364640	-0.2662660	-0.3055710
**30**	C	5.9641290	-1.6728510	-0.8923320
**31**	H	6.9877270	-1.9549270	-0.5954150
**32**	H	6.0333470	-1.2715150	-1.9153210
**33**	N	5.1721210	-2.9046100	-0.9470420
**34**	C	7.5113920	0.5336210	0.8021430
**35**	H	8.1134280	-0.3271320	0.4725870
**36**	H	8.1273380	1.4297940	0.6318100
**37**	C	5.0888070	-3.6797030	0.2876560
**38**	H	5.8423140	-3.3260600	1.0053010
**39**	H	4.1064490	-3.6189180	0.7877090
**40**	C	3.9985810	-2.9429470	-1.8036620
**41**	H	3.7132710	-3.9940080	-1.9644840
**42**	H	4.2474270	-2.5186090	-2.7879860
**43**	O	2.1918000	-2.0859100	0.2493160
**44**	O	1.3502880	-2.9193390	-2.1059470
**45**	H	0.3972730	-2.7481210	-1.8340660
**46**	O	4.6352940	-6.1287690	-1.0754500
**47**	O	5.4243160	-6.0328160	1.4576620
**48**	H	5.5062010	-7.0069090	1.4781650
**49**	O	6.3973030	1.2607050	3.3445210
**50**	O	8.9224420	0.5672700	3.1869650
**51**	H	9.0872100	1.4895300	3.4651260
**52**	P	7.3892180	0.3841420	2.6506480
**53**	P	5.4750100	-5.4394140	-0.0478020
**54**	P	2.4419040	-2.1054610	-1.2292920
**55**	O	2.5620820	-0.6318430	-1.9032500
**56**	H	1.9821080	0.0601060	-1.4934900
**57**	O	7.0674400	-5.4961380	-0.3869350
**58**	H	7.2103770	-5.6321020	-1.3433900
**59**	O	7.1933620	-1.2151170	2.8470300
**60**	H	6.9898650	-1.4471860	3.7757520
**61**	Ag	-3.3386250	0.8635100	-2.1931270
**62**	Ag	-4.1554470	-1.3689970	-0.6652900
**63**	Ag	-2.2435890	1.6410370	0.3188270
**64**	Ag	-2.2771300	-2.5676420	1.0323620
**65**	Ag	-0.7843390	-0.2415590	1.7980900
**66**	Ag	-1.3529720	-0.6890420	-0.9395460
**67**	Ag	-5.0499830	1.2017620	0.0707160
**68**	Ag	-3.6190040	-0.3042830	1.9714460
**69**	Ag	-2.0379650	-3.2447280	-1.7145790

**70**	Ag	-4.0296080	2.5425080	2.2949810

Table S10. Geometrical parameters of DTPMP model adsorption of N-Ag_{10}.

Number	Element	NA	NB	NC	Bond (Å)	Angle (º)	Dihedral (º)
**1**	C						
**2**	H	1			1.1071532		
**3**	H	1	2		1.0992266	107.1697045	
**4**	N	1	3	2	1.4705956	106.4182016	120.9703671
**5**	C	4	1	3	1.4518802	117.7685837	175.8460998
**6**	H	5	4	1	1.1006325	109.3427837	-171.4204756
**7**	H	5	4	1	1.1011830	110.1747328	-55.5075487
**8**	C	4	1	5	1.4606478	114.7243625	141.2902275
**9**	H	8	4	1	1.1030962	113.7044322	-112.9934778
**10**	H	8	4	1	1.0997261	110.5409834	5.8874557
**11**	P	8	4	1	1.8349885	114.2192699	125.1203629
**12**	O	11	8	4	1.4946121	113.8510903	175.4062347
**13**	P	5	4	1	1.8565995	121.3141789	66.1614675
**14**	O	13	5	4	1.4949230	113.4303824	178.9827402
**15**	O	11	8	4	1.6231214	102.6932877	-56.7044012
**16**	H	15	11	8	1.0098330	112.5569441	177.6926874
**17**	O	11	8	4	1.6311882	106.9902294	49.1382888
**18**	H	17	11	8	0.9774519	109.9965166	105.1859301
**19**	O	13	5	4	1.6209985	102.1485850	51.7723295
**20**	H	19	13	5	0.9786443	112.6750178	-172.2006572
**21**	O	13	5	4	1.6324825	108.7496962	-54.1657292
**22**	H	21	13	5	0.9774320	110.4141477	-101.3922074
**23**	C	1	4	5	1.5367027	112.8325669	57.3757283
**24**	H	23	1	4	1.1074915	109.5851150	38.5670941
**25**	H	23	1	4	1.0991632	110.3481690	-79.0441431
**26**	N	23	1	4	1.4723790	109.7275380	162.0280314
**27**	C	26	23	1	1.4734174	113.0136158	-80.8849345
**28**	H	27	26	23	1.1076486	111.6985901	-88.6246214
**29**	H	27	26	23	1.0968817	107.4564657	27.9601386
**30**	C	27	26	23	1.5423339	112.0944030	147.1016577
**31**	H	30	27	26	1.1024878	109.9006540	58.4737722
**32**	H	30	27	26	1.1010759	108.8642399	-57.8930110
**33**	N	30	27	26	1.4654351	116.0298711	-179.6517682
**34**	C	26	23	1	1.4531941	114.9207438	143.9558400
**35**	H	34	26	23	1.1008861	110.6958137	-169.3331502
**36**	H	34	26	23	1.1006947	109.4551212	-52.2233511
**37**	C	33	30	27	1.4602019	116.4513866	-64.4511908
**38**	H	37	33	30	1.0990225	110.0121136	-13.5518159
**39**	H	37	33	30	1.1039815	113.8627561	106.8638651
**40**	C	33	30	27	1.4534317	118.7217135	81.7347192
**41**	H	40	33	30	1.1009063	108.6950946	162.8997227

**42**	H	40	33	30	1.1004003	109.5386480	46.0420968
**43**	O	40	33	30	2.8659534	94.5280470	-91.6179327
**44**	O	43	40	33	2.6362872	57.7729250	-171.8211803
**45**	H	44	43	40	1.0057199	90.4089548	-178.8783083
**46**	O	37	33	30	2.8393056	93.4987384	-153.8366039
**47**	O	37	33	30	2.6492680	146.8958337	-126.1585116
**48**	H	47	37	33	0.9777437	154.7914081	-21.7105605
**49**	O	34	26	23	2.8694131	97.4692647	49.5885177
**50**	O	49	34	26	2.6233572	60.4014282	176.5133210
**51**	H	50	49	34	0.9772848	84.0156272	115.6515585
**52**	P	49	34	26	1.4945627	35.1498248	143.1088021
**53**	P	46	37	33	1.4954577	35.1942930	128.0034517
**54**	P	43	40	33	1.4997386	35.3856826	152.3040370
**55**	O	54	43	40	1.6248730	114.1698652	-119.6387499
**56**	H	55	54	43	0.9914971	114.7234796	-34.2080217
**57**	O	53	46	37	1.6291292	112.9397387	-123.4231424
**58**	H	57	53	46	0.9765876	110.5968991	23.2369349
**59**	O	52	49	34	1.6231312	116.2073228	-120.9277893
**60**	H	59	52	49	0.9786683	111.9344128	-43.4234081
**61**	Ag	12	11	8	4.3701441	107.9797263	151.9752782
**62**	Ag	61	12	11	2.8258754	139.5728329	-17.2695357
**63**	Ag	61	12	11	2.8484320	70.0446731	30.4827353
**64**	Ag	62	61	12	2.8012224	118.1573106	-1.3513069
**65**	Ag	63	61	12	2.8038897	118.8195336	-104.1486574
**66**	Ag	63	61	12	2.7939165	59.8479485	-88.7414975
**67**	Ag	62	61	12	2.8196988	60.8268460	104.8377993
**68**	Ag	64	62	61	2.7937964	62.2891175	82.6372397
**69**	Ag	66	63	61	2.7570677	145.1327822	-68.1073492
**70**	Ag	67	62	61	2.7903619	122.0141762	-106.0011839

a)

b)

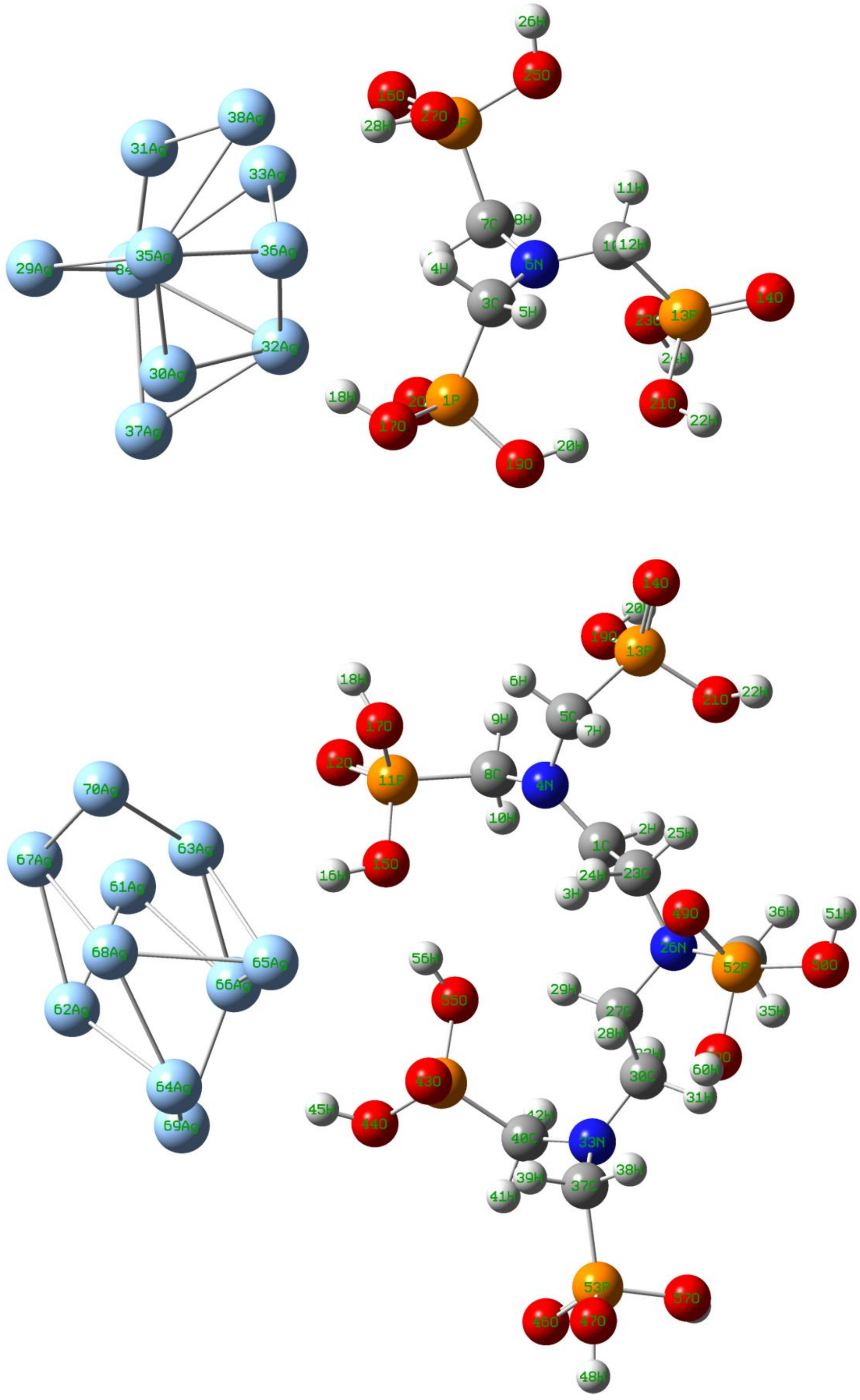

Figure S7. DFT optimized structure of a) ATMP and b) DTPMP with a model of absorption Ag_{10}-phosphonate group.

Table S11. Complete theoretical (BPV86/6–311 +G(2d.p)) and experimental SERS wavenumbers (λ_0 = 785 nm) of ATMP (1000 ppm / 3.44 × 10^{-3} mol L^{-1}), with the respectives assignments based on the potential energy distribution computed with the VEDA 4xx software.

Theoretical ATMP SERS wavenumbers (cm^{-1}) (DFT/BPV86)	Experimental ATMP SERS wavenumbers (cm^{-1}) (1000 ppm / 3.34 × 10^{-3} mol L^{-1}) (λ_0 = 785 nm)	Assigments[a]
3336	-	$\nu(O_{27}H_{28})$ (100%)
3319	-	$\nu(O_{19}H_{20})$ (100%)
3148	-	$\nu(O_{17}H_{18})$ (100%)
3024	-	$\nu(C_7H_8)$ (38%) + $\nu(C_7H_9)$ (28%) + $\nu(C_{10}H_{11})$ (19%) + $\nu(C_{10}H_{12})$ (14%)
2974	-	$\nu(C_7H_8)$ (28%) + $\nu(C_7H_9)$ (39%) + $\nu(C_{10}H_{11})$ (14%)+ $\nu(C_{10}H_{12})$ (19%)
2868	-	$\nu(C_3H_4)$ (97%)
1418	1422 w	$\delta(H_4C_3H_5)$ (19%) + $\delta(H_9C_7H_8)$ (38%) + $\delta(H_{12}C_{10}H_{11})$ (15%)
1385	-	$\delta(H_4C_3H_5)$ (24%) + $\delta(H_{12}C_{10}H_{11})$ (49%)
1347	1313 w	$\nu(N_6C_{10})$ (12%) + $\nu(N_6C_3)$ (11%) + $\delta(H_5C_3N_6)$ (10%) + $\delta(H_8C_7N_6)$ (35%)
1323	-	$\nu(N_6C_7)$ (16%) + $\delta(H_5C_3N_6)$ (19%) + $\delta(H_{11}C_{10}N_6)$ (30%)
1289	-	$\delta(H_{12}C_{10}H_{11})$ (11%) + $\tau(H_{11}C_{10}N_6C_7)$ (27%) + $\tau(H_{12}C_{10}N_6C_7)$ (27%)
1266	-	$\delta(H_9C_7H_8)$ (12%) + $\tau(H_8C_7N_6C_{10})$ (29%) + $\tau(H_9C_7N_6C_{10})$ (26%) + $\tau(H_{11}C_{10}N_6C_7)$ (10%)
1255	-	$\delta(H_4C_3H_5)$ (15%) + $\tau(H_4C_3N_6C_7)$ (44%) + $\tau(H_5C_3N_6C_7)$ (23%)
1196	-	$\nu(P_{13}O_{14})$ (68%)
1186	-	$\nu(P_{13}O_{14})$ (11%) + $\delta(H_5C_3N_6)$ (23%) + $\delta(H_8C_7N_6)$ (24%) + $\delta(H_{11}C_{10}N_6)$ (11%)
1162	-	$\delta(H_{20}O_{19}P_1)$ (77%)
1141	-	$\nu(P_1O_2)$ (18%) + $\nu(P_{15}O_{16})$ (51%)
1133	-	$\nu(P_1O_2)$ (51%) + $\nu(P_{15}O_{16})$ (26%)
1118	-	$\delta(H_{11}C_{10}N_6)$ (16%) + $\nu(N_6C_3)$ (20%) + $\nu(N_6C_7)$ (26%)
1104	-	$\nu(N_6C_3)$ (12%) + $\nu(N_6C_{10})$ (29%) + $\delta(H_5C_3N_6)$ (14%)
1075	1051 sh	$\delta(H_{18}O_{19}P_1)$ (87%)
1037	-	$\delta(H_{22}O_{21}P_{13})$ (79%)
1015	1027 s	$\delta(H_{28}O_{27}P_{15})$ (48%) + $\delta(H_{26}O_{25}P_{15})$ (34%)
1003	973 s	$\delta(H_{28}O_{27}P_{15})$ (25%) + $\delta(H_{26}O_{25}P_{15})$ (54%)
877	-	$\nu(P_1O_{19})$ (24%)
855	-	$\nu(P_1O_{19})$ (30%)
844	849 sh	$\nu(N_6C_3)$ (10%) + $\nu(N_6C_7)$ (14%) + $\nu(N_6C_{10})$ (11%) + $\nu(P_1O_{17})$ (31)
822	-	$\nu(P_1O_{19})$ (20%) + $\nu(P_1O_{17})$ (35%) + $\nu(P_{13}O_{23})$ (14%)
804	822 w	$\nu(N_6C_3)$ (10%) + $\nu(P_{13}O_{23})$ (32%) + $\nu(P_{15}O_{25})$ (16%)
783	-	$\nu(P_{15}C_{25})$ (35%)
764	-	$\nu(P_{13}O_{21})$ (54%) + $\nu(P_{13}C_{10})$ (11%)
675	770 s	$\nu(P_1C_3)$ (52%) + $\delta(P_1C_3N_6)$ (13%)
659	712 vs	$\nu(P_{13}O_{21})$ (24%) + $\nu(P_{13}C_{10})$ (42%)

648	-	ν($P_{15}O_{27}$) (12%) + ν($P_{15}C_7$) (50%) + δ($P_{15}C_7N_6$) (12%)
579	-	τ($H_{20}O_{19}P_1Ag_{32}$) (84%)
491	550 m	τ($H_{18}O_{17}P_1Ag_{32}$) (78%)
448	460 w	τ($H_{28}O_{27}P_{15}Ag_{33}$) (57%)
410	-	δ($C_3N_6C_{10}$) (23%) + δ($O_{19}P_1O_2$) (14%) + δ($O_{25}P_{15}O_{16}$) (16%)
379	-	δ($O_{19}P_1O_2$) (26%) + δ($O_{25}P_{15}O_{16}$) (25%)
356	-	δ($O_{27}P_{15}O_{16}$) (55%)
321	-	δ($O_{16}P_{15}C_7$) (10%)
309	-	τ($H_{22}O_{21}P_{13}C_{10}$) (18%) + γ($O_{14}C_{10}O_{23}P_{13}$) (13%)
282	-	γ($O_{17}Ag_{32}O_2P_1$) (10%) + τ($H_{22}O_{21}P_{13}C_{10}$) (41%)
258	-	δ($C_3P_1O_2$) (20%)
236	-	τ($H_{26}O_{25}P_{15}Ag_{33}$) (42%)
218	-	τ($H_{26}O_{25}P_{15}Ag_{33}$) (17%) + τ($H_{24}O_{23}P_{13}C_{10}$) (36%)
192	-	δ($C_7N_6C_3$) (12%) + δ($C_3P_1O_2$) (11%) + γ($O_{25}Ag_{33}O_{16}P_{15}$) (12%)

ν, stretching; δ, in-plane deformation; τ, torsional; γ, out-of-plane.

v. very; s. strong; m. medium; w. weak; sh. shoulder.

[a]All assignments include internal coordinates that contribute 10% or more to the PED.

Table S12. Complete theoretical (BPV86/6–311 +G(2d.p)) and experimental SERS wavenumbers (λ_0 = 785 nm) of DTPMP (1000 ppm / 1.74 × 10^{-3} mol L^{-1}), with the respective assignments based on the potential energy distribution computed with the VEDA 4xx software.

Theoretical DTPMP SERS wavenumbers (cm^{-1}) (DFT/BPV86)	Experimental DTPMP SERS wavenumbers (cm^{-1}) (1000 ppm / 1.74 × 10^{-3} mol L^{-1}) (λ_0 = 785 nm)	Assigments[a]
3692	-	ν($O_{57}H_{58}$) (100%)
3680	-	ν($O_{21}H_{22}$) (96%)
3657	-	ν($O_{19}H_{20}$) (100%)
3386	-	ν($O_{55}H_{56}$) (99%)
3055	-	ν($O_{44}H_{45}$) (99%)
3026	-	ν(C_5H_6) (49%) + ν(C_5H_7) (30%)
2996	-	ν($O_{15}H_{16}$) (96%)
2975	-	ν($C_{34}H_{35}$) (55%) + ν($C_{34}H_{36}$) (44%)
2955	-	ν($C_{30}H_{31}$) (68%) + ν($C_{30}H_{32}$) (22%)
2900	-	ν(C_1H_2) (27%) + ν($C_{23}H_{24}$) (70%)
1441	1447 m	δ($H_2C_1H_3$) (31%) + δ($H_{29}C_{27}H_{28}$) (20%) + δ($H_{32}C_{30}H_{31}$) (20%)
1429	-	δ($H_{29}C_{27}H_{28}$) (18%) + δ($H_{32}C_{30}H_{31}$) (45%)
1417	-	δ($H_7C_5H_6$) (13%) + δ($H_{10}C_8H_9$) (53%) +

1405	-	$\delta(H_{39}C_{37}H_{38})$ (60%) + $\delta(H_{32}C_{30}H_{31})$ (13%)
1389	-	$\delta(H_{42}C_{40}H_{41})$ (13%) + $\delta(H_{36}C_{34}H_{35})$ (54%)
1371	-	$\tau(H_{29}C_{27}N_{26}C_{23})$ (23%) + $\tau(H_{28}C_{27}N_{26}C_{23})$ (16%) + $\delta(H_{29}C_{27}H_{28})$ (13%)
1331	-	$\delta(H_{41}C_{40}N_{33})$ (42%)
1321	-	$\delta(H_{31}C_{30}N_{33})$ (11%) + $\delta(H_{35}C_{34}N_{26})$ (10%)
1303	-	$\delta(H_3C_1N_4)$ (13%) + $\delta(H_{24}C_{23}C_1)$ (20%) + $\delta(H_{35}C_{34}N_{26})$ (11%)
1281	-	$\delta(H_{42}C_{40}H_{41})$ (12%) + $\tau(H_{41}C_{40}N_{33}C_{30})$ (28%) + $\tau(H_{42}C_{40}N_{33}C_{30})$ (27%) +
1267	-	$\tau(H_{35}C_{34}N_{26}C_{23})$ (30%) + $\tau(H_{36}C_{34}N_{26}C_{23})$ (38%) + $\delta(H_{28}C_{27}C_{30})$ (18%)
1260	-	$\tau(H_{38}C_{37}N_{33}C_{30})$ (26%) + $\tau(H_{39}C_{37}N_{33}C_{30})$ (34%) + $\delta(H_{39}C_{37}H_{38})$ (11%)
1177	-	$\nu(P_{13}O_{14})$ (21%)
1166	-	$\nu(P_{54}O_{43})$ (55%)
1154	-	$\nu(P_{54}O_{43})$ (18%) + $\delta(H_{38}C_{37}N_{33})$ (12%)
1113	-	$\nu(N_4C_5)$ (19%)
1092	-	$\delta(P_{11}O_{15}H_{16})$ (33%) + $\delta(H_{56}O_{55}P_{54})$ (31%)
1045	1046 m	$\delta(H_{56}O_{55}P_{54})$ (33%) + $\delta(P_{11}O_{15}H_{16})$ (17%) + $\delta(H_{45}O_{44}P_{54})$ (17%) +
1028	-	$\nu(N_{33}C_{37})$ (10%) + $\nu(N_{30}C_{40})$ (10%) + $\delta(H_{31}C_{30}N_{33})$ (13%)
1011	951 s	$\delta(H_{45}O_{44}P_{54})$ (58%) + $\delta(H_{18}O_{17}P_{11})$ (11%) + $\delta(H_{56}O_{55}P_{54})$ (17%)
833	-	$\nu(P_{11}O_{17})$ (21%) +
821	-	$\nu(N_{33}C_{37})$ (10%) + $\nu(N_{33}C_{30})$ (14%)
813	-	$\nu(P_{53}O_{47})$ (39%) + $\nu(P_{54}O_{44})$ (13%) + $\nu(P_{53}O_{57})$ (18%)
807	-	$\nu(P_{11}O_{17})$ (18%)
791	-	$\nu(P_{53}O_{47})$ (26%) + $\nu(P_{53}O_{57})$ (31%)
781	-	$\nu(P_{54}O_{44})$ (20%) + $\nu(P_{54}O_{55})$ (19%)
691	-	$\nu(P_{53}C_{37})$ (46%) + $\delta(P_{53}C_{37}N_{33})$ (12%) + $\nu(P_{53}O_{57})$ (14 %)
680	765 s	$\nu(P_{11}O_{15})$ (13%) + $\nu(P_{11}O_{17})$ (16%) + $\nu(P_{11}C_8)$ (40%)
651	718 m	$\nu(P_{52}C_{34})$ (43%) + $\delta(P_{52}C_{34}N_{26})$ (22%)
638	-	$\nu(P_{13}O_{21})$ (13%) + $\nu(P_{13}C_5)$ (44%) + $\delta(P_{13}C_5N_4)$ (16%)
574	536 w	$\tau(H_{56}O_{55}P_{54}C_{40})$ (69%)
548	-	$\nu(P_{52}C_{34})$ (13%)
533	-	$\delta(C_{23}C_1N_4)$ (10%)

494	-	δ($P_{54}C_{40}N_{33}$) (11%)
483	-	τ($H_{56}O_{55}P_{54}C_{40}$) (13%) + τ($H_{45}O_{44}P_{54}C_{40}$) (13%)
456	448 m	δ($O_{44}P_{54}O_{43}$) (11%) + τ($H_{45}O_{44}P_{54}C_{40}$) (61%)
412	-	δ($C_{40}N_{33}C_{30}$) (18%) + δ($O_{46}P_{53}O_{57}$) (11%)
397	-	δ($O_{19}P_{13}O_{21}$) (16%) + τ($H_{22}O_{21}P_{13}C_{5}$) (17%)
376	-	δ($O_{43}P_{54}O_{55}$) (38%)
362	-	δ($O_{55}P_{54}O_{44}$) (46%) +
286	-	τ($P_{53}O_{57}O_{47}C_{37}$) (12%)
274	-	τ($P_{52}O_{59}O_{47}C_{37}$) (11%) + τ($P_{53}O_{57}O_{47}C_{37}$) (12%)
247	-	τ($H_{18}O_{17}P_{11}C_{8}$) (17%) + τ($H_{51}O_{50}P_{52}C_{34}$) (11%) + τ($H_{60}O_{59}P_{52}C_{34}$) (25%)
239	-	δ($O_{44}P_{54}O_{43}$) (10%) + τ($H_{48}O_{47}P_{53}C_{37}$) (12%) + τ($P_{54}O_{43}O_{55}C_{40}$) (14%)
229	-	τ($H_{18}O_{17}P_{11}C_{8}$) (29%)
213	-	τ($H_{48}O_{47}P_{53}C_{37}$) (11%) + τ($P_{54}O_{43}O_{55}C_{40}$) (24%)
191	-	τ($H_{48}O_{47}P_{53}C_{37}$) (23%)

ν, stretching; δ, in-plane deformation; τ, torsional;

v. very; s. strong; m. medium; w. weak; sh. shoulder.

[a]All assignments include internal coordinates that contribute 10% or more to the PED.
